# Lipid saturation controls nuclear envelope function

**DOI:** 10.1038/s41556-023-01207-8

**Published:** 2023-08-17

**Authors:** Anete Romanauska, Alwin Köhler

**Affiliations:** 1 Max Perutz Labs, Vienna Biocenter Campus (VBC), Vienna, Austria; 2 Center for Molecular Biology, University of Vienna, Vienna, Austria; 3 Center for Medical Biochemistry, Medical University of Vienna, Vienna, Austria

**Keywords:** Nuclear envelope, Nuclear pore complex

## Abstract

The nuclear envelope (NE) is a spherical double membrane with elastic properties. How NE shape and elasticity are regulated by lipid chemistry is unknown. Here we discover lipid acyl chain unsaturation as essential for NE and nuclear pore complex (NPC) architecture and function. Increased lipid saturation rigidifies the NE and the endoplasmic reticulum into planar, polygonal membranes, which are fracture prone. These membranes exhibit a micron-scale segregation of lipids into ordered and disordered phases, excluding NPCs from the ordered phase. Balanced lipid saturation is required for NPC integrity, pore membrane curvature and nucleocytoplasmic transport. Oxygen deprivation amplifies the impact of saturated lipids, causing NE rigidification and rupture. Conversely, lipid droplets buffer saturated lipids to preserve NE architecture. Our study uncovers a fundamental link between lipid acyl chain structure and the integrity of the cell nucleus with implications for nuclear membrane malfunction in ischaemic tissues.

## Abstract (web summary)

Romanauska and Köhler manipulate the levels of endogenously produced saturated acyl chains in yeast and show that nuclear envelope and nuclear pore complex function are uniquely sensitive to lipid acyl chain unsaturation.

## Main

Key properties of a biological membrane include elasticity, fluidity, phase behaviour, thickness and curvature, all of which depend on both lipids and lipid-interacting proteins^1–3^. The nuclear envelope (NE) is a specialized double membrane enabling eukaryotic cells to protect and regulate their genome. The NE connects to the endoplasmic reticulum (ER) by NE–ER junctions that allow lipid exchange^4^. The NE comprises an inner nuclear membrane (INM) and outer nuclear membrane (ONM), which are fused at highly curved membrane pores, into which nuclear pore complexes (NPCs) are embedded^5^. NPCs are transport channels composed of nucleoporins (Nups), which in yeast form a 52 MDa membrane-anchored assembly^6–10^. NPCs interact with the pore membrane through transmembrane proteins and amphipathic helices^6,11–14^. NPCs experience deformations of the NE and respond by pore channel dilation and constriction^6,15^, which may alter nuclear mechanotransduction^16,17^ and nucleocytoplasmic transport^18^. Altered nuclear elasticity can lead to NE herniations, rupture and genome instability^19–22^. Despite this advancement, we do not understand how NE lipids contribute to NE elasticity and how they influence NPCs.

Cellular phospholipids differ in their polar headgroups, and in length and unsaturation level of their fatty acyl chains, which can range from saturated (only C–C bonds) to monounsaturated (one C=C bond) to polyunsaturated^23^. Most eukaryotes contain a ∆9-desaturase (yeast Ole1, human SCD1), which introduces a C=C bond and kink in the acyl chain^24^. Yeast Ole1 is subject to feedback control that measures the ratio of saturated to unsaturated fatty acids (SFAs/UFAs)^25,26^. Ole1 competes with the glycerol-3-phosphate acyltransferase (GPAT) Sct1 for the common substrate C16:0-CoA. Sct1 sequesters C16:0-CoA into lipids thereby preventing desaturation by Ole1. Exogenous SFAs inhibit Sct1, shifting the balance towards Ole1’s desaturase activity^27^. Failure to maintain a balanced SFA/UFA ratio can lead to lipotoxicity^28,29^.

Elasticity and bending rigidity of a membrane determine the forces that have to be applied to produce deviations from the spontaneous shape^30^. Lipids with long SFAs make membranes thicker, less elastic and less fluid because of tighter packing of their hydrophobic tails and stronger lateral lipid interactions^31^. Unsaturated lipids do the opposite because kinked acyl chains prevent tight packing. In artificial membranes, unsaturated lipids, saturated lipids and cholesterol separate laterally into liquid phases, generating regions with high lipid packing (liquid-ordered domains; L_o_) and less lipid packing (liquid-disordered domains; L_d_)^32–34^. Cholesterol interacts more favourably with saturated than with unsaturated lipids, thus preferring the L_o_ phase. The L_o_ phase is more rigid than the L_d_ phase, at least in vitro^32,35^, and does not promote the formation of highly curved structures. L_o_/L_d_ formation has been extensively studied in vitro, but analysing lipid domains in live cells remains a challenge^36–40^. The NE requirements for lipid (un)saturation, lipid order/disorder and membrane elasticity are unknown. With few exceptions^41,42^, even the lipid composition of the NE remains undetermined. In this Article, we developed tools to address these questions in *Saccharomyces cerevisiae* cells, which revealed that finely balanced lipid saturation is critical to maintain NE/ER architecture and function.

## Results

### GPAT enzymes rigidify NE and ER membranes

**Fig. 1:**
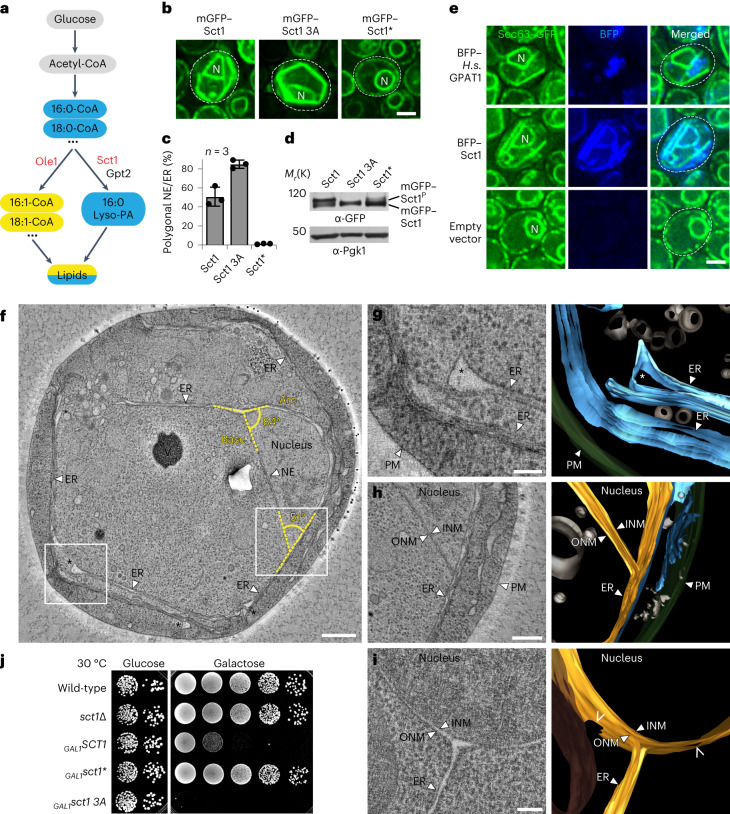
GPATs regulate NE/ER membrane morphology. **a**, Cartoon of the competition between Ole1 and Sct1 for C16:0-CoA. **b**, Live imaging of *sct1*Δ cells overexpressing plasmid-based mGFP-*SCT1* constructs. Sct1 3A, Sct1 S664A S668A S671A. Sct1*, catalytically inactive mutant Sct1 G253L. Dashed white line marks cell contours. N, nucleus. Scale bar, 2 µm. **c**, Quantification of the prevalence of a polygonal NE/ER in **b**. Mean value and standard deviation indicated. *n*, number of biological replicates; >350 cells analysed for each condition. **d**, Immunoblotting for Sct1 phosphorylation. Samples taken from cultures used in **b** and analysed on 3–8% Tris–acetate gel in Tris–acetate SDS buffer. Top band: phosphorylated Sct1; lower band: unphosphorylated Sct1. Pgk1 (3-phosphoglycerate kinase) as loading control. **e**, Imaging of cells expressing genomically tagged *SEC63*–GFP as ER marker and empty vector or plasmid-based, overexpressed *S.c. SCT1* or *H.s. GPAT1* tagged with BFP. N, nucleus. Scale bar, 2 µm. **f**–**h**, TEM and 3D reconstructions of the NE/ER in Sct1-overexpressing cell (**f**), showing membrane blistering (white asterisks) at the edges of ER sheets (**g**) and an NE–ER connection with an acute angle (**h**) (boxed areas in **f**). For an animated 3D model, see Supplementary Video 1. V, vacuole. Scale bar, 500 nm (**f**), 200 nm (**h**) or 100 nm (**g**). **i**, TEM and 3D reconstruction of NE–ER connection in wild-type cell. For an animated 3D model, see Supplementary Video 2. Open arrowheads indicate pore membranes. Scale bar, 100 nm. **j**, Growth analysis of wild-type or *sct1*Δ cells transformed with plasmid-based constructs overexpressed from the galactose-inducible *GAL1* promoter. Growth on plates containing glucose (repressed) or galactose (induced) at 30 °C. Source numerical data and unprocessed blots are available in source data. Source data

**Extended Data Fig. 1:**
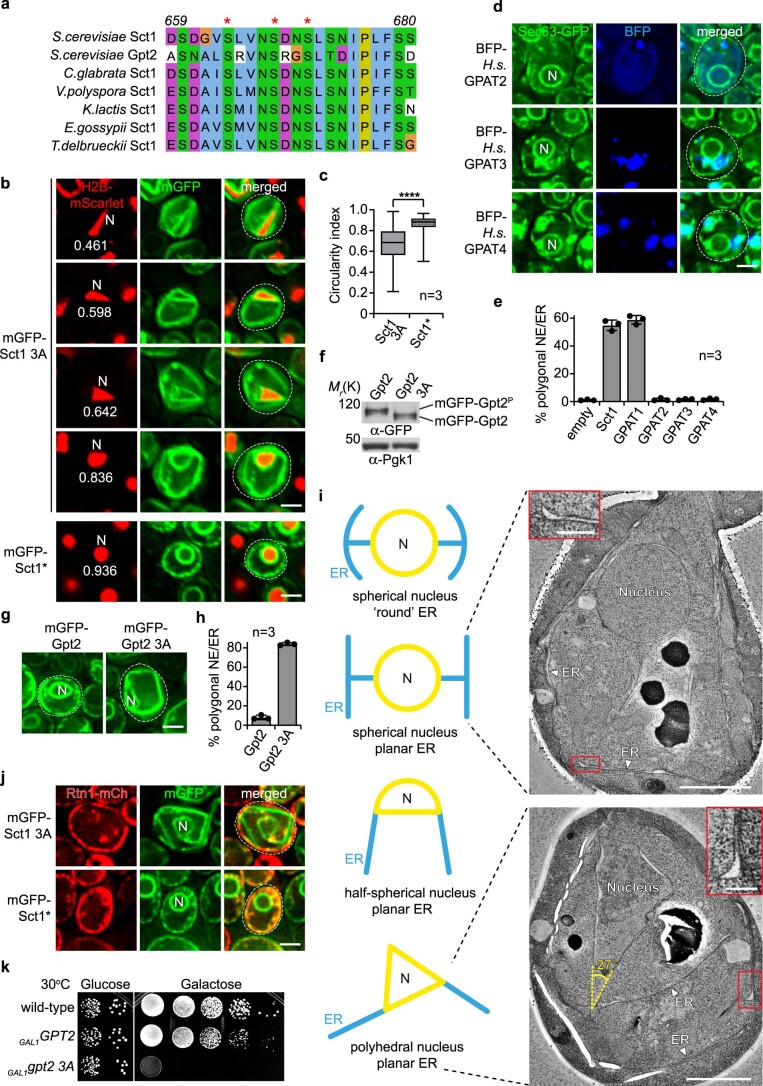
GPATs regulate NE/ER membrane morphology. **a**, Sequence alignment of a conserved phosphorylation motif located at the C-termini of Sct1 and Gpt2. Red asterisks: serine residues that were mutated to alanine in Sct1 3A or Gpt2 3A. Amino acid positions in Sct1 indicated. **b**, Live imaging of *sct1*Δ cells expressing genomically tagged *H2B*-mScarlet and overexpressing plasmid-based mGFP*-SCT1* constructs. Numbers: circularity index of H2B signal, taken as a proxy for nuclear shape. Circularity of Sct1 3A-expressing cells is significantly reduced, consistent with NE rigidification. Sct1* = Sct1 G253L. N, nucleus. Scale bar, 2 µm. **c**, Quantification of circularity index of nuclear H2B signal in (b). Value of 1 indicates a perfect circle. As the value approaches 0, the structure becomes increasingly irregular. p-value (****<0.0001) determined by two-sided Mann-Whitney test. Box-plot indicates median and interquartile range, whiskers extend to the smallest and largest value. n = number of biological replicates; >450 cells analyzed for each condition. **d**, Imaging of cells expressing genomically tagged *SEC63*-GFP and overexpressing plasmid-based *Homo sapiens* (*H.s*.) *GPAT* constructs tagged with BFP. N, nucleus. Scale bar, 2 µm. **e**, Prevalence of polygonal NE/ER phenotype in (d and Fig. 1e). Mean value and standard deviation indicated. n = number of biological replicates; >300 cells analyzed for each condition. **f**, Immunoblotting for Gpt2 phosphorylation. Samples analyzed on a 3–8% Tris-acetate gel in Tris-acetate SDS buffer. Upper band: phosphorylated Gpt2; lower band: unphosphorylated Gpt2. Gpt2 3A = Gpt2 S664A S668A S671A. Pgk1 (3-phosphoglycerate kinase) as loading control. **g**, Imaging of *gpt2*Δ cells overexpressing plasmid-based mGFP-*GPT2* constructs. N, nucleus. Scale bar, 2 µm. **h**, Prevalence of polygonal NE/ER phenotype in (g). Mean value and standard deviation indicated. n = number of biological replicates; >500 cells analyzed for each condition. **i**, TEM analysis of *sct1*Δ cells overexpressing plasmid-based mGFP-*SCT1* from the *GAL1* promoter. Insets show a magnified view of marked areas. Schemes illustrate the most frequently observed NE/ER morphologies in cells with increased lipid saturation. Hypothetically, the order of phenotypes (from top to bottom) reflects the evolution of membrane rigidification, starting with the ER (spherical nucleus/planar ER) and progressing through one stiff NE membrane (half-spherical nucleus/planar ER; see Fig. 1f) to two or more stiff NE membranes (polyhedral nucleus/planar ER). ER, endoplasmic reticulum. Scale bar, 1 µm; 200 nm for insets. **j**, Imaging of *sct1*Δ cells expressing plasmid-based mGFP-*SCT1* constructs and the reticulon *RTN1*-mCherry. Constructs were overexpressed from the *GAL1* promoter. Note that Rtn1 does not overlap with rigidified ER sheets. Sct1* = Sct1 G253L. N, nucleus. Scale bar, 2 µm. **k**, Growth of wild-type cells overexpressing plasmid-based constructs from the *GAL1* promoter. Empty vector used as control. Growth on plates containing glucose (repressed) and galactose (induced). Source numerical data and unprocessed blots are available in source data. Source data

**Extended Data Fig. 2:**
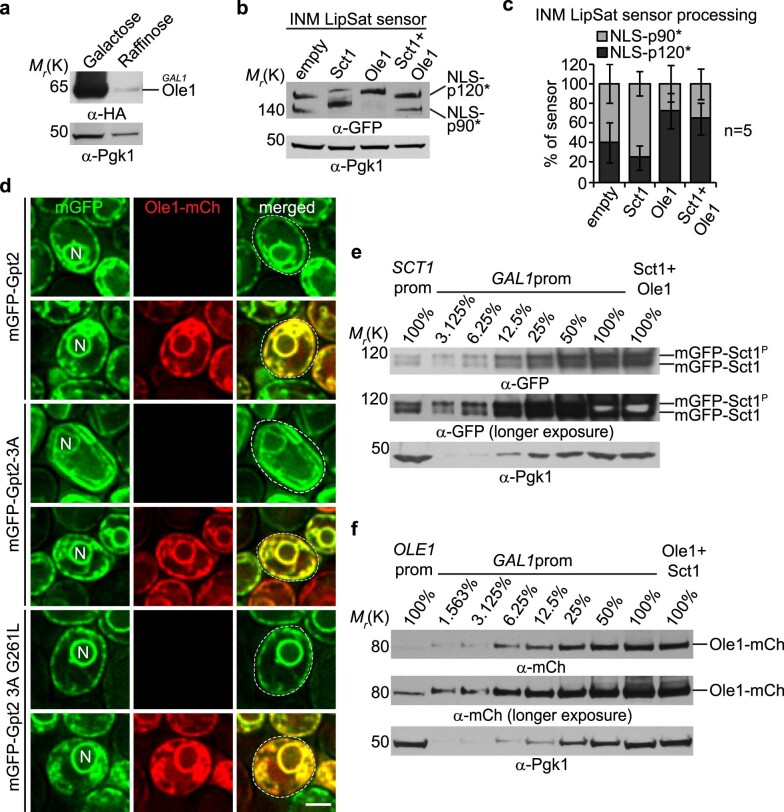
Ole1 counteracts GPAT-induced NE/ER rigidification. **a**, Immunoblotting of Ole1 overexpressed from a *GAL1* promoter. Samples from cultures used in (Fig. 2a) and analyzed on a 12% Bis-Tris gel in MOPS SDS buffer. Pgk1 (3-phosphoglycerate kinase) as loading control. **b**, Immunoblotting of INM LipSat sensor processing. Samples taken from cultures used in (Fig. 2d) and analyzed on a 12% Bis-Tris gel in MOPS SDS buffer. NLS-p120*: membrane bound form of the INM LipSat sensor; NLS-p90*: processed, soluble form. NLS corresponds to the Heh2 aa93–317 sequence, which was appended to the LipSat sensor for nuclear import. Pgk1 (3-phosphoglycerate kinase) as loading control. **c**, Quantification of INM LipSat sensor processing in (b). Percentage of NLS-p120* and NLS-p90* relative to the total amount of sensor. Mean value and standard deviation indicated. n = number of biological replicates. **d**, Live imaging of *gpt2*Δ cells overexpressing plasmid-based mGFP-*GPT2* constructs and/or *OLE1*-mCherry. Gpt2 G261L is a catalytically inactive mutant. N, nucleus. Scale bar, 2 µm. **e**, Quantitative immunoblotting of Sct1, expressed from the endogenous *SCT1* promoter, overexpressed from the *GAL1* promoter, or co-overexpressed with *OLE1* in *sct1*Δ cells. Sct1 was overexpressed ~20 fold compared to endogenous protein levels, and Ole1 co-expression did not noticeably alter Sct1 protein levels. Denaturing extracts were prepared and analyzed on a 3–8% Tris-acetate gel in Tris-acetate SDS buffer. Serial dilutions of cell extracts shown. Upper band: phosphorylated Sct1; lower band: unphosphorylated Sct1. Pgk1 (3-phosphoglycerate kinase) as loading control. **f**, Quantitative immunoblotting of Ole1, expressed from the endogenous *OLE1* promoter, overexpressed from the *GAL1* promoter, or co-overexpressed with *SCT1*. Ole1 was overexpressed ~100 fold compared to endogenous protein levels, and Sct1 co-expression did not noticeably alter Ole1 protein levels. Denaturing extracts were prepared and analyzed on a 12% Bis-Tris protein gel in MOPS SDS buffer. Serial dilutions of cell extracts shown. Pgk1 (3-phosphoglycerate kinase) as loading control. Source numerical data and unprocessed blots are available in source data. Source data

Acyl chain profiles have been manipulated by exogenous fatty acid overload, but any conclusions are compromised by rapid fatty acid turnover and side effects of the overload^29^. To avoid this, we altered the level of endogenously produced saturated acyl chains. The GPAT Sct1 incorporates C16:0 acyl chains into glycerolipids, thereby preventing them from desaturation by Ole1 (Fig. 1a) and increasing the ratio of saturated over unsaturated lipids^27^. Taking advantage of these observations, we overexpressed Sct1, tagged with monomeric green fluorescent protein (mGFP), from an inducible *GAL1* promoter. Because Sct1 is embedded in the ER membrane, the mGFP tag allowed us to visualize both the induction and the morphology of the NE and ER. Unexpectedly, the normally spherical nucleus was transformed into an irregular polygon with straight sides of different length and different angles in cross-section (Fig. 1b and Extended Data Fig. 1b,c). The ER was also transformed into straight membranes with edges (Fig. 1b). Thus, increased GPAT activity induces a striking rigidification of the NE/ER network. The phenotype was present in ∼50% of cells (Fig. 1c). Sct1 was overexpressed ~20 fold compared with endogenous levels (Extended Data Fig. 2e), which is expected to increase the overall content of saturated acyl chains from ∼12 to ∼40% (ref. ^27^). Phosphorylation of Sct1 inhibits its enzymatic activity^27^. Preventing this inhibition by mutating three conserved phosphorylation sites to alanine (Sct1 3A) (Extended Data Fig. 1a) raised the penetrance of NE/ER rigidification to ∼80% (Fig. 1b–d). Polygonal NE/ER membranes were caused by Sct1 enzymatic activity, because overexpressing a catalytically inactive mutant (Sct1 G253L, termed Sct1*) (ref. ^27^) yielded a regular NE/ER morphology (Fig. 1b–d). The ability of GPAT enzymes to rigidify the NE and ER may be conserved and isoform specific, since overexpressing human GPAT1 in yeast elicited a similar membrane geometry (Fig. 1e), whereas other human GPATs did not (Extended Data Fig. 1d,e). Rat GPAT1 also prefers saturated over unsaturated fatty acyl-CoAs^43^, suggesting conserved substrate specificity. Yeast has two GPATs, Sct1 and Gpt2. Gpt2 was phosphorylated and hence probably inactive. When we mutated its three putative phosphorylation sites to alanine (Gpt2 3A)^44^ (Extended Data Fig. 1a,f), overexpression also rigidified the NE/ER in ∼80% of cells (Extended Data Fig. 1g,h). Thus, yeast and human GPATs can stiffen the NE and ER.

### Extensive membrane planarity in the NE/ER network

To study the ultrastructure of rigidified NE/ER membranes, we employed transmission electron microscopy (TEM) and 3D reconstruction. In cross-section, increased GPAT activity induced nuclei with typically one or more straight membranes (Fig. 1f and Extended Data Fig. 1i). By 2D TEM, the NE frequently appeared as a semicircle with a straight ‘base’ covered by an ‘arc’, presumably a 3D hemisphere (Fig. 1f and Supplementary Video 1). A planar ER, often consisting of stacks of ER membrane (Fig. 1f), was found in conjunction with either a spherical nucleus (Extended Data Fig. 1i), a half-spherical nucleus (Fig. 1f) or a nucleus that appeared to consist entirely of planar membranes (approximating a polyhedron, Extended Data Fig. 1i), suggesting that rigidification may progress in this order. NE–ER connections are rare in wild-type cells (approximately eight per nucleus)^4^ and often emanate from the NE as cisternae in a perpendicular orientation (Fig. 1i and Supplementary Video 2). In contrast, high GPAT activity induced wide NE–ER connections that were a continuous extension of the NE hemisphere (Fig. 1h). The edges of ER sheets typically appeared ‘blistered’, probably because rigid membranes do not tolerate high membrane curvature (Fig. 1g). ER curvature and shape are normally stabilized by reticulons such as Rtn1 (ref. ^4^). However, overexpressing Rtn1 together with Sct1 did not prevent ER rigidification, possibly because Rtn1 failed to bind to ER sheets enriched in saturated lipids (Extended Data Fig. 1j). NE/ER rigidification correlated with a substantial cell growth defect (Fig. 1j and Extended Data Fig. 1k). Hence, a stiffened NE/ER may contribute to the cellular lipotoxicity that was described in human cells treated with SFAs^45^.

### Rigid NE/ER membranes are caused by lipid saturation

**Fig. 2:**
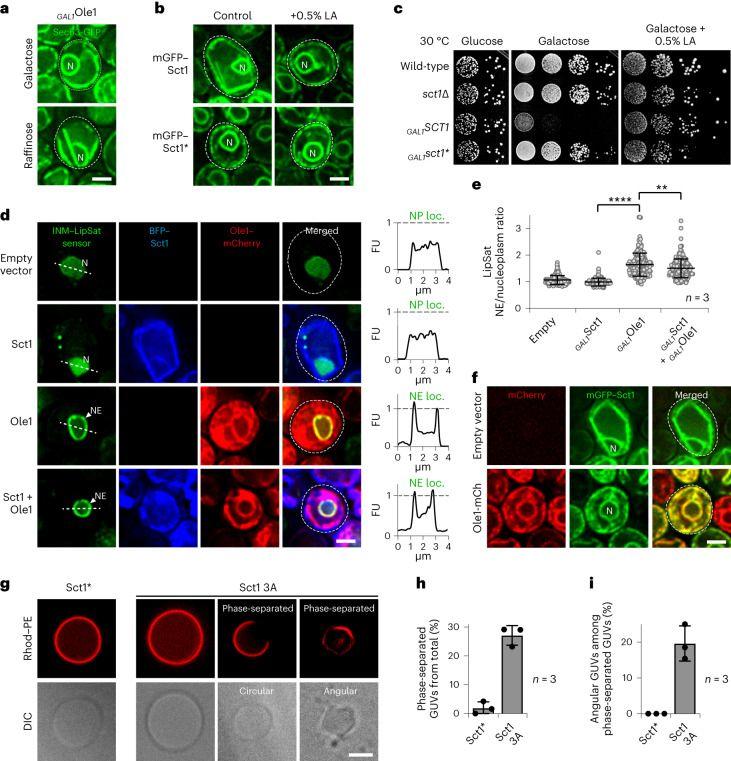
NE/ER rigidity is caused by increased lipid saturation. **a**, Live imaging of _*GAL1*_*OLE1* cells expressing genomically tagged *SEC63*–GFP. Growth on medium containing galactose (highly expressed *OLE1*) or raffinose (lowly expressed *OLE1*). N, nucleus. Scale bar, 2 µm. **b**, Imaging of *sct1*Δ cells overexpressing plasmid-based mGFP–*SCT1* constructs. Cells were grown for 2 h in galactose-containing medium, then treated with 0.5% LA (+1% Tween 80) for 2 h. Sct1*, Sct1 G253L. Scale bar, 2 µm. **c**, Growth analysis of wild-type or *sct1*Δ cells transformed with plasmid-based constructs overexpressed from the *GAL1* promoter. Plates contained glucose (repressed) or galactose (induced), or galactose supplemented with 0.5% LA (+1% Tween 80). **d**, Imaging of *sct1*Δ cells expressing a plasmid-based INM LipSat sensor^46^ and *SCT1* or *OLE1* overexpressed from a *GAL1* promoter. Sensor fluorescence intensity was measured across a line spanning the nucleus. FU, arbitrary fluorescence units; N, nucleus; NP loc., nucleoplasmic localization; NE loc., nuclear envelope localization. The FU value of 1 is marked with a dashed line. Scale bar, 2 µm. **e**, Quantification of INM LipSat sensor distribution in **d**. Ratios of NE and nucleoplasmic fluorescence intensities were calculated. Mean value and standard deviation indicated. *P* values (**P = 0.0011, ****P < 0.0001) determined by two-sided Mann–Whitney test. *n*, number of biological replicates; >170 cells analysed for each condition. **f**, Imaging of *sct1*Δ cells containing an empty vector or expressing plasmid-based mGFP–*SCT1* and *OLE1*–mCherry from a *GAL1* promoter. Scale bar, 2 µm. **g**, GUV reconstitution from whole cell lipid extracts from the indicated Sct1-overexpressing strains. Rhod–PE prefers the liquid-disordered phase. Scale bar, 4 µm. **h**, Quantification of phase-separated GUVs in **g**. Mean value and standard deviation indicated. *n*, number of biological replicates; >190 GUVs analysed for each condition. **i**, Quantification of angular GUVs among phase-separated GUVs. Mean value and standard deviation indicated. *n*, number of biological replicates; >190 GUVs analysed for each condition. Source numerical data are available in source data. Source data

To verify that the flattening of NE/ER membranes is caused by increased lipid saturation, we conditionally inactivated Ole1, the antagonist of Sct1 (Fig. 1a). Ole1 depletion recapitulated the effect of Sct1 overexpression and rigidified NE/ER membranes (Fig. 2a and Extended Data Fig. 2a). Thus, a balance of Ole1 desaturase and Sct1 GPAT activity is essential to prevent rigidification of the NE and ER. This led us to predict that exogenously supplemented linoleic acid (LA), a polyunsaturated FA, would cure Sct1-induced NE/ER rigidification. Indeed, LA reversed membrane stiffening and Sct1 toxicity (Fig. 2b,c). To directly confirm that Sct1 overexpression increases the amount of saturated lipids in the NE/ER network, we used a previously developed lipid saturation (LipSat) sensor^46^ that measures the lipid acyl chain saturation state of the INM. When membranes contain high levels of saturated lipids, the mGFP-tagged sensor is proteolytically processed and released from the INM, resulting in a nucleoplasmic fluorescent signal. In contrast, the LipSat sensor remains INM-bound when saturated lipids are low. Sct1 activity led to a nucleoplasmic LipSat sensor localization, confirming high levels of saturated lipids at the INM and probably the entire NE/ER^46^ (Fig. 2d,e and Extended Data Fig. 2b,c). Consistent with the substrate competition between Sct1 and Ole1 (Fig. 1a), co-overexpression of Ole1 with Sct1 decreased the levels of saturated INM-lipids (Fig. 2d,e and Extended Data Fig. 2b,c) and reversed the rigidity of NE/ER membranes (Fig. 2f and Extended Data Fig. 2d–f).

To further explore how lipid saturation shapes NE/ER geometry, we asked whether membrane rigidification can be reconstituted in a simplified in vitro system. We isolated lipids from Sct1-overexpressing cells and generated giant unilamellar vesicles (GUVs) from them. Notably, ~27% of GUVs showed hallmarks of lipid phase separation, characterized by the lateral segregation of lipids into liquid-ordered (L_o_) and liquid-disordered (L_d_) domains (Fig. 2g,h)^47^. Strikingly, ~20% of phase-separated GUVs exhibited angular shapes in cross-section (Fig. 2g,i), reminiscent of the polygonal NE structures observed in cells (Fig. 1b). In contrast, lipids extracted from cells overexpressing the inactive Sct1 variant (Sct1*) produced only circular GUVs, with ~2% exhibiting phase-separation. Thus, we find both in vivo and in vitro evidence that rigid NE/ER membranes are caused by lipid saturation. Lipid phase separation may play a key role in this process (see further below).

### Lipid saturation regulates nuclear pore function

**Fig. 3:**
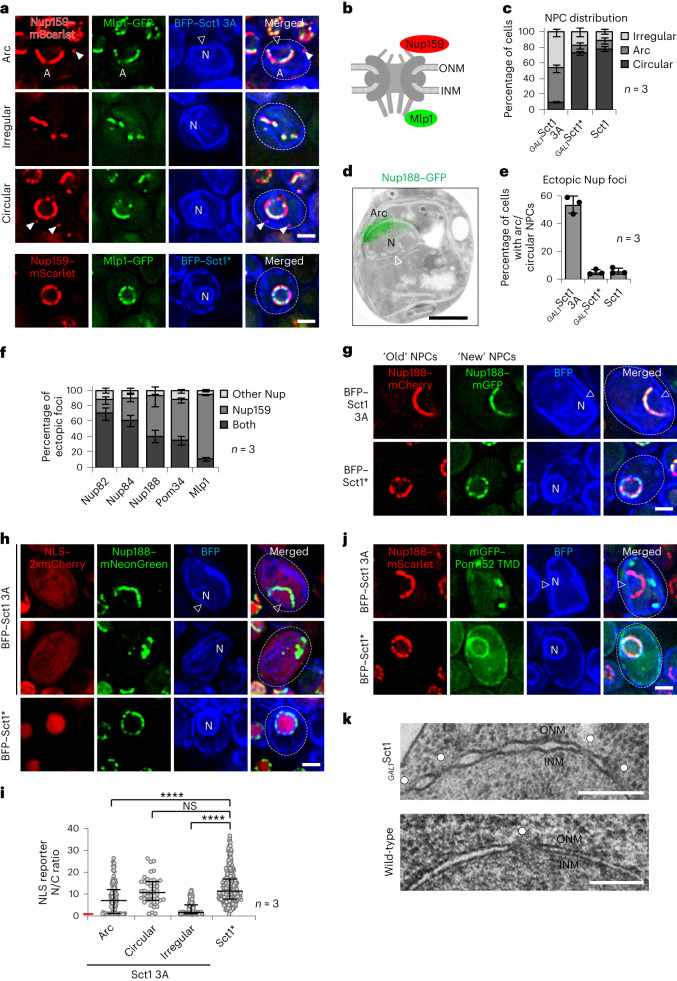
Lipid saturation affects NPC distribution, integrity and function. **a**, Live imaging of *sct1*Δ cells expressing genomically tagged *NUP159*–mScarlet, *MLP1*–GFP and plasmid-based, overexpressed BFP*–SCT1* constructs. White arrowheads: ectopic Nup foci; empty arrowhead: straight NE ‘base’ covered by NPC ‘arc’. Sct1*, Sct1 G253L; N, nucleus; A, ‘arc’. Scale bar, 2 µm. **b**, Cartoon of Nup localization in **a**. **c**, Quantification of NPC distribution in **a**, classified as ‘circular’, ‘irregular’ or ‘arc’. Mean value and standard deviation indicated. *n*, number of biological replicates; >600 cells analysed for each condition. **d**, Correlative light and electron microscopy of Nup188–GFP in Sct1 3A-overexpressing cells. Empty arrowhead: ‘base’. Scale bar, 1 µm. **e**, Prevalence of ectopic Nup foci in cells with ‘arc’ or ‘circular’ NPC phenotypes as shown in **a**. Mean value and standard deviation indicated. *n*, number of biological replicates; >350 cells analysed for each condition. **f**, Quantification of ectopic Nup foci composition in **a** and Extended Data Fig. 3a–e. Mean value and standard deviation indicated. *n*, number of biological replicates; >275 foci analysed for each condition. **g**, Imaging of *sct1*Δ cells expressing inducible Cre recombinase and *NUP188* genomically tagged with the RITE cassette. Plasmid-based BFP–*SCT1* constructs were overexpressed. Images recorded 4 h after Cre induction. Empty arrowhead: straight NE ‘base’. Scale bar, 2 µm. **h**, Imaging of *sct1*Δ cells expressing plasmid-based NLS–2xmCherry, genomically tagged *NUP188*–mNeonGreen and overexpressing BFP–*SCT1* constructs. Examples of ‘arc’ and ‘irregular’ NPC patterns are shown. Empty arrowhead: straight NE ‘base’. NLS, nuclear localization sequence. Scale bar, 2 µm. **i**, Quantification of nuclear import efficiency in **h**. The ratio of NLS–2xmCherry fluorescence intensity in the nucleus and cytoplasm (N/C) was calculated. NPC phenotypes were classified as ‘circular’, ‘irregular’ or ‘arc’. Median and interquartile range indicated. *P* value (****P < 0.0001) determined by two-sided Mann–Whitney test. NS, not significant (*P* = 0.254). An N/C ratio of ‘1’ (red line on *y* axis) indicates breakdown of nuclear import. *n*, number of biological replicates; >340 cells analysed for Sct1 3A and Sct1* cells, respectively. **j**, Imaging of *sct1*Δ cells expressing genomically tagged *NUP188*–mScarlet, plasmid-based mGFP*–POM152* TMD and overexpressing BFP–*SCT1* constructs. Empty arrowhead: straight NE ‘base’. Scale bar, 2 µm. **k**, TEM examples of NE blistering upon mGFP–*SCT1* overexpression. Wild-type cells used as control. White circles: pore membranes. Scale bar, 200 nm. Source numerical data are available in source data. Source data

**Extended Data Fig. 3:**
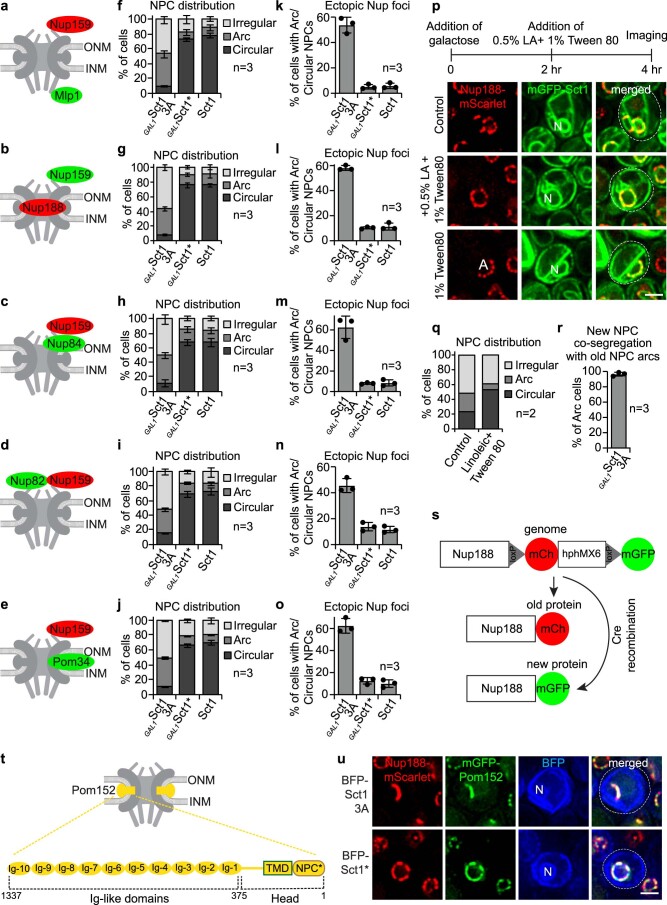
Lipid saturation regulates nuclear pore distribution and assembly. **a-e**, Cartoons of nucleoporin localization (that is cytoplasmic side, NPC core, or NPC basket) for microscopy experiments (see also Fig. 3a, b). Cartoons refer to graphs on the right side. INM, inner nuclear membrane; ONM, outer nuclear membrane. **f-j**, Quantification of NPC distribution shows similar patterns irrespective of the chosen nucleoporins. NPC distributions classified as ‘circular’, ‘irregular’, or ‘arc’. Mean value and standard deviation indicated. n = number of biological replicates; >600 cells analyzed for each condition. Sct1* = Sct1 G253L. **k-o**, Prevalence of ectopic nucleoporin foci in cells with ‘arc’ or ‘circular’ NPC phenotypes. Mean value and standard deviation indicated. n = number of biological replicates; >295 cells analyzed for each condition. **p**, Live imaging of *sct1*Δ cells expressing genomically tagged *NUP188*-mScarlet and overexpressing plasmid-based mGFP-*SCT1*. Cells were grown for 2 h in galactose-containing medium, then treated with 0.5% linoleic acid (LA, +1% Tween 80) for 2 h or treated only with 1% Tween 80 for 4 h in galactose-containing medium. N, nucleus. Scale bar, 2 µm. **q**, Quantification of NPC distribution in (p). Phenotypes classified as ‘circular’, ‘irregular’, or ‘arc’. Mean value and standard deviation indicated. n = number of biological replicates; >330 cells analyzed for each condition. **r**, Quantification of co-segregation of new NPCs with old NPC ‘arcs’ in (Fig. 3g). Mean value and standard deviation indicated. n = number of biological replicates; 195 cells analyzed. **s**, Cartoon of recombination-induced tag exchange (RITE) experiment. After induction of Cre recombinase, mCherry fluorescent tag of *NUP188* is switched to mGFP. **t**, Cartoon of Pom152 position in the NPC and its domain organization. Numbers indicate Pom152 amino acids. INM, inner nuclear membrane; ONM, outer nuclear membrane; TMD, transmembrane domain; NPC*, domain that faces the NPC transport channel; Ig, immunoglobulin-like domain, located in the perinuclear space. **u**, Imaging of *sct1*Δ cells expressing genomically tagged *NUP188*-mScarlet, plasmid-based full-length mGFP-*POM152*, and overexpressing BFP-*SCT1* constructs. N, nucleus. Scale bar, 2 µm. Source numerical data are available in source data. Source data

**Extended Data Fig. 4:**
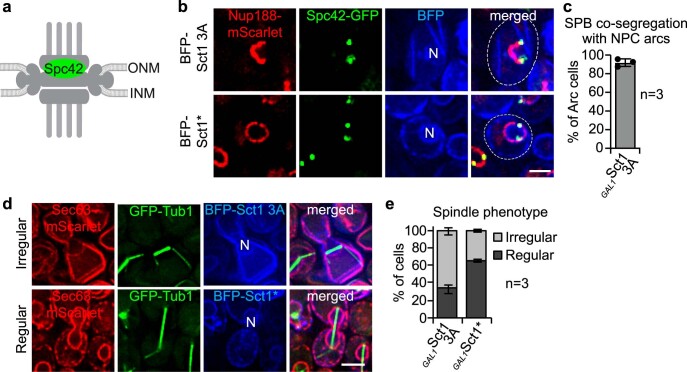
Increased lipid saturation affects spindle pole body function. **a**, Cartoon of NE-embedded spindle pole body (SPB) and its Spc42 subunit, tagged in (b). INM, inner nuclear membrane; ONM, outer nuclear membrane. **b**, Live imaging of *sct1*Δ cells expressing genomically tagged *NUP188*-mScarlet, *SPC42*-GFP, and overexpressing plasmid-based BFP*-SCT1* constructs. Sct1* = Sct1 G253L. N, nucleus. Scale bar, 2 µm. **c**, Quantification of spindle pole body (SPB) co-segregation with NPC ‘arcs’ in (b). Note that SPBs localize to outer ends of an ‘arc’ in 64% of cases analyzed. Mean value and standard deviation indicated. n = number of biological replicates; 352 cells analyzed. **d**, Live imaging of *sct1*Δ cells expressing genomically tagged *SEC63*-mScarlet, integrated GFP-*TUB1*, and overexpressing plasmid-based BFP-*SCT1* constructs. Cells were synchronized with α-factor. N, nucleus. Scale bar, 2 µm. **e**, Quantification of spindle phenotypes in (d). Phenotypes classified as regular or irregular. Mean value and standard deviation indicated. n = number of biological replicates; >200 spindles analyzed for each condition. Source numerical data are available in source data. Source data

To understand how lipid saturation impinges on NE-resident membrane proteins, we examined the NPC as the largest membrane-tethered assembly of the NE/ER. We visualized a Nup on the cytoplasmic side (Nup159–mScarlet, a cytoplasmic filament Nup) and one on the nucleoplasmic side (Mlp1–GFP, a basket Nup) (Fig. 3b and Extended Data Fig. 3a–e). These Nups exhibited a circular NE distribution in wild-type cells or cells overexpressing catalytically inactive Sct1 (Fig. 3a, bottom). Strikingly, increased lipid saturation caused a large-scale segregation of NPCs on the NE. Approximately 90% of cells exhibited NPC ‘arcs’ or ‘irregular’ NPC patterns, the latter of which probably represent scattered NPC-like assemblies in ER membranes (Fig. 3a,c and Extended Data Fig. 3f–j). Only ~10% of cells displayed a normal circular NPC pattern on the NE. By correlative light and electron microscopy, the NPC ‘arc’ co-localized with the NE ‘arc’ that sits atop a straight NE ‘base’ (Fig. 3d). In contrast, the NE ‘base’ was depleted of NPCs, suggesting major differences in the material properties of these distinct NE territories. Confirming increased lipid saturation as the root cause of NPC segregation, the addition of polyunsaturated LA partially reversed the phenotypes (Extended Data Fig. 3p,q). Additionally, upon increased lipid saturation, we observed ectopic, smaller Nup foci outside of the ‘arc’ or ‘circular’ NPC distribution in ~50% of cells (Fig. 3e and Extended Data Fig. 3k–o). To assess whether these ectopic foci contain intact NPCs, we co-localized Nup159 with Nups in structural proximity (Nup82), the adjacent Y-complex (Nup84), the inner ring complex (IRC; Nup188), transmembrane Nups (Pom34) and the nuclear pore basket (Mlp1) on the opposite side of the NPC (Fig. 3f and Extended Data Fig. 3a–e). Only ~10% of ectopic NPCs contained the topologically outermost Nups, Nup159 and Mlp1, together; >50% of foci lacked IRC and transmembrane Nups and to a lesser extent the Y-complex (Fig. 3f). This hints to defective NPC assembly or stability caused by increased lipid saturation. To test whether new NPCs still assemble, we employed recombination-induced tag exchange (RITE)^48^. New and old NPCs were differentially labelled by inducing genetic recombination to change a red (mCherry) to a green (mGFP) fluorescent tag on Nup188 (Extended Data Fig. 3s) concomitant with the induction of rigid NE/ER membranes. New NPCs did assemble and preferentially inserted into the NE ‘arc’, avoiding the straight NE ‘base’ (Fig. 3g and Extended Data Fig. 3r). This indicates that the curved ‘arc’ membrane is more permissive for NPC biogenesis than the rigid ‘base’. We then assessed whether NPCs in a rigidified NE were functional by measuring nuclear import with an NLS–2xmCherry reporter. Cell nuclei with a ‘circular’ NPC pattern exhibited a nuclear to cytoplasmic (N/C) ratio of the reporter that was similar to control cells (Sct1*). In contrast, the N/C ratio of the reporter in cells with NPC ‘arcs’ was lower, consistent with a partial loss of import competence. The N/C ratio in cells with ‘irregular’ NPC patterns was further reduced, and its median approached the value of 1. This suggests a breakdown of nuclear import, a loss of NE integrity or a combination of both (Fig. 3h,i). We conclude that high levels of saturated lipids impair NPC assembly and function. In sum, a balanced lipid saturation is essential for the architectural integrity, correct distribution and transport function of NPCs. Yeast spindle pole bodies, the functional equivalent of mammalian centrosomes, are embedded in the NE via transmembrane proteins (for example Ndc1, also present in NPCs). When examining SPBs via Spc42–GFP in cells with NPC ‘arcs’, ~90% of SPBs were found in the ‘arc’—typically at the edge of the ‘arc’, presumably because spindle forces maximize the distance between the two SPBs (Extended Data Fig. 4a–c). Defective SPB positioning translated into aberrant mitotic spindles (Extended Data Fig. 4d,e). This indicates that SPBs, like NPCs, require a balanced lipid saturation for their proper positioning and function.

### Lipid saturation controls pore membrane architecture

Membrane protein interactions with the lipid bilayer require a hydrophobic match between transmembrane domain (TMD) length and lipid bilayer thickness^49^. In general, saturated lipids produce thicker membranes than unsaturated lipids with equal carbon atom number. The proteinaceous ring that encircles the NPC in the lumen of the NE consists mainly of Pom152 in yeast^6^. Pom152 harbours a TMD inserted into the pore membrane. Pom152 self-oligomerizes through immunoglobulin-like repeats within the NE lumen (Extended Data Fig. 3t)^6,50,51^. To study the impact of lipid saturation, we compared the behaviour of full-length Pom152 with the Pom152 TMD. Full-length mGFP–Pom152 co-localized with the IRC subunit Nup188–mScarlet under conditions of low or high lipid saturation (Extended Data Fig. 3u). In contrast, the Pom152 TMD diffused freely throughout the NE/ER when lipid saturation was low, consistent with the removal of its NPC-interacting parts outside of the TMD. Notably, raising lipid saturation caused an aggregation of the Pom152 TMD into multiple foci that did not co-localize with NPC ‘arcs’ (Fig. 3j). This suggests that high levels of saturated lipids perturb the insertion or stability of the Pom152 TMD. Hence, even when this key TMD Nup is held in place by protein–protein interactions, its membrane insertion could be perturbed. Importantly, we frequently observed a ‘blistered’ NE around NPCs (Fig. 3k). This geometry indicates a reduced pore membrane curvature in oversaturated NEs and may mechanistically resemble the ‘blistered’, less curved edges of ER membranes (Fig. 1g). Our results provide cellular evidence for in vitro findings that membranes rich in saturated lipids are less elastic and do not favour the formation of highly curved structures^32,35^. This situation conflicts with the proper embedding of transmembrane Nups and NPC assembly.

### Balanced lipid saturation prevents lipid phase separation

**Fig. 4:**
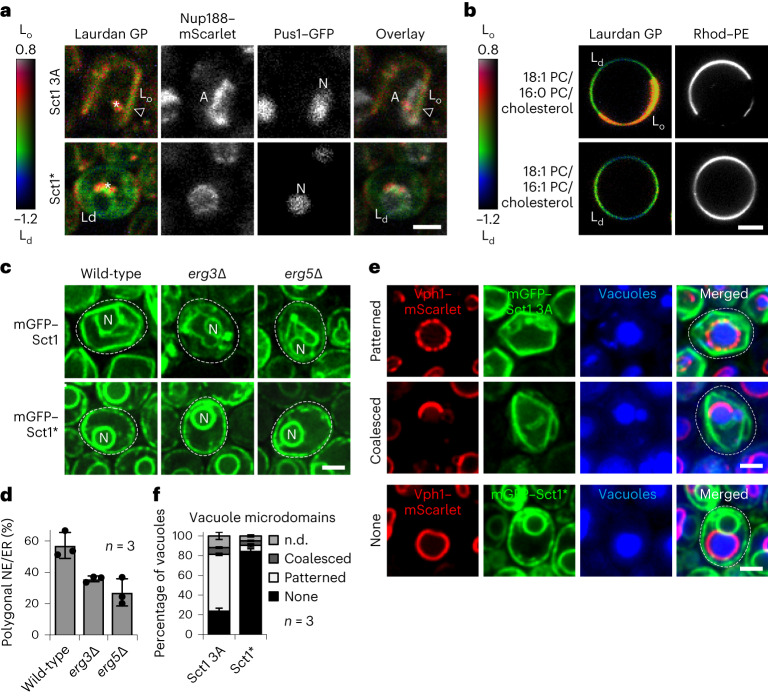
Increased lipid saturation triggers NE/ER lipid phase separation. **a**, Single-plane, pseudocoloured generalized polarization (GP) images of Laurdan-stained cells. Colour bar designates range of GP values. Red indicates the highest and blue the lowest membrane order. *sct1*Δ cells expressed genomically tagged *NUP188*–mScarlet, the nucleoplasmic marker *PUS1*–GFP and overexpressed plasmid-based *SCT1* constructs. Empty arrowhead: straight NE ‘base’. Note that the ‘arc’ side of the NE is less ordered than the opposite NE membrane. White asterisks: LDs, which accumulate Laurdan due to their apolar interior^76^. Sct1*, Sct1 G253L; L_d_, liquid-disordered phase; L_o_, liquid-ordered phase; A, ‘arc’; N, nucleus. Scale bar, 2 µm. **b**, Single-plane, GP images of GUVs stained with Laurdan and Rhod–PE. Rhod–PE partitions into the liquid-disordered (L_d_) phase. GUVs were prepared from 18:1 PC/16:0 PC/cholesterol or 18:1 PC/16:1 PC/cholesterol lipid mixes in 1:2:0.25 ratio. L_o_, liquid-ordered phase. Scale bar, 5 µm. **c**, Imaging of *erg3*Δ, *erg5*Δ or wild-type cells overexpressing plasmid-based mGFP–*SCT1* constructs. Representative examples of *erg3*Δ and *erg5*Δ cells do not exhibit a polygonal NE/ER. Sct1 overexpression increases cellular membrane content^27^, which may lead to a curled appearance of the NE/ER network upon *erg3*Δ and *erg5*Δ deletion. N, nucleus; Sct1*, Sct1 G253L. Scale bar, 2 µm. **d**, Quantification of the prevalence of a polygonal NE/ER in **c**. Mean value and standard deviation indicated. *n*, number of biological replicates; >450 cells analysed for each condition. **e**, Imaging of cells expressing genomically tagged *VPH1*–mScarlet and overexpressing plasmid-based mGFP–*SCT1* constructs. Vacuoles stained with CellTracker Blue. Sct1*, Sct1 G253L. Scale bar, 2 µm. **f**, Quantification of vacuole microdomains in **e**. Phenotypes classified as ‘patterned’, ‘coalesced’, ‘none’ or ‘n.d.’ (not determinable). Mean value and standard deviation indicated. *n*, number of biological replicates; >340 vacuoles analysed for each condition. Source numerical data are available in source data. Source data

**Extended Data Fig. 5:**
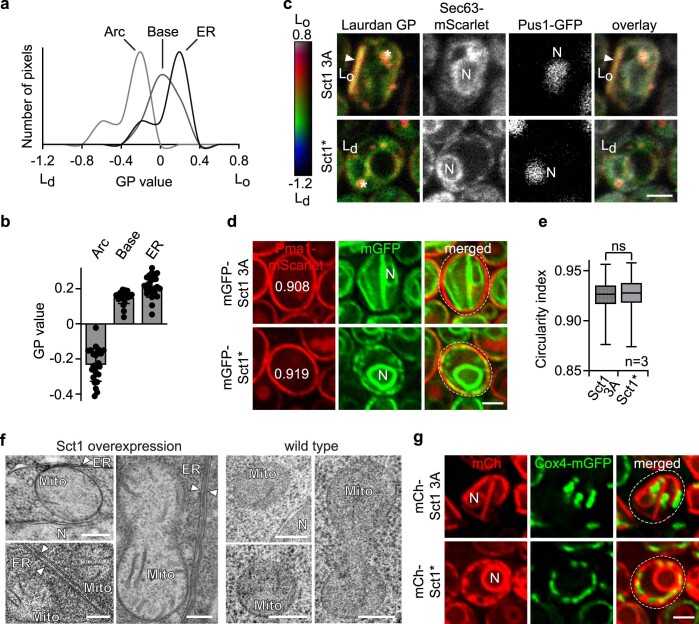
Plasma membrane and mitochondria responses to lipid saturation stress. **a**, Generalized polarization (GP) values of NE ‘arc’, NE ‘base’, and peripheral ER regions in a cell overexpressing Sct1 3A in (Fig. 4a). Note that the GP curves for the ER and the NE ‘base’ are shifted to the right, indicating a higher membrane order than the NE ‘arc’. **b**, GP values of NE ‘arc’, ‘base’, and ER regions in cells overexpressing Sct1 3A. Mean value and standard deviation indicated. 25 ‘arc’ cells analyzed. **c**, Single-plane, pseudocolored generalized polarization (GP) images of Laurdan-stained cells. Color bar designates the range of GP values. Red indicates the highest, blue the lowest membrane order. *sct1*Δ cells expressed genomically tagged *SEC63*-mScarlet, the nucleoplasmic marker *PUS1*-GFP, and overexpressed plasmid-based *SCT1* constructs. White arrowhead highlights the lack of co-localization between the highly ordered ER membrane and the Sec63 translocon. White asterisks: lipid droplets, which accumulate Laurdan due to their apolar interior^76^. Sct1* = Sct1 G253L. L_d_, liquid-disordered phase; L_o_, liquid-ordered phase; N, nucleus. Scale bar, 2 µm. **d**, Live imaging of *sct1*Δ cells expressing genomically tagged *PMA1*-mScarlet and overexpressing plasmid-based mGFP*-SCT1* constructs. Numbers: circularity index of the Pma1 fluorescence signal, taken as a proxy for the plasma membrane contour. Sct1* = Sct1 G253L. N, nucleus. Scale bar, 2 µm. **e**, Quantification of circularity index of Pma1 contour in (d). Circularity index of 1 indicates a perfect circle. ns (not significant, p-value 0.205) as determined by two-sided Mann-Whitney test. Box-plot indicates median and interquartile range, whiskers extend to the smallest and largest value. n = number of biological replicates; >250 cells analyzed. **f**, TEM images of mitochondria in cells overexpressing mGFP-*SCT1* from the *GAL1* promoter. Wild-type cells used as a control. Arrows label rigidified ER membranes. Mito, mitochondrion; N, nucleus; ER, endoplasmic reticulum. Scale bar, 200 nm. **g**, Imaging of *sct1*Δ cells expressing genomically tagged mitochondrial marker *COX4*-mGFP and overexpressing plasmid-based mCherry*-SCT1* constructs. Sct1* = Sct1 G253L. N, nucleus. Scale bar, 2 µm. Source numerical data are available in source data. Source data

The segregation of NPCs into NE ‘arcs’ in the presence of high lipid saturation raises the question whether this is caused by an underlying segregation of lipids^32–34^. To test this, we employed Laurdan (6-lauryl-2-dimethylamino-naphtalene), a polarity-sensitive membrane probe^52^. The less polar environment of a liquid-ordered L_o_ phase shifts the emission spectrum compared with that in a liquid-disordered L_d_ phase, allowing a quantitative assessment of membrane order. We simultaneously visualized L_o_/L_d_ phases by Laurdan staining, NPCs via Nup188–mScarlet, and the nucleoplasm via the enzyme Pus1–GFP (Fig. 4a). Using multi-photon microscopy, we observed a micron-scale segregation of NE/ER lipids into L_o_ and L_d_ phases when cellular lipid saturation was raised. Overall, the membrane order in the ER and the ‘base’ of the NE was increased, consistent with the rigidification of these membranes (Fig. 1f). The NE ‘arc’ region that co-localizes with NPCs exhibited a largely disordered lipid state compared with the NE ‘base’ and the peripheral ER (Fig. 4a and Extended Data Fig. 5a,b). Control cells exhibited an evenly disordered membrane state in both the NE and ER. The localization of NPCs in an L_d_ territory indicates that the liquid-disordered phase is more permissive for accommodating NPCs (Fig. 3g). A tendency to segregate into the L_d_ phase was also observed for the ER translocon (Sec63–mScarlet) (Extended Data Fig. 5c). We recapitulated lipid phase separation in a simplified in vitro system. A ternary mixture of saturated lipids (16:0 phosphatidylcholine, a product of Sct1 activity in vivo), unsaturated lipids (18:1 phosphatidylcholine) and cholesterol led to lipid demixing into hemispheres of L_d_ and L_o_ phases, detectable by Laurdan and rhodamine–PE. The effect was not seen when 16:0 phosphatidylcholine was replaced by 16:1 phosphatidylcholine (Fig. 4b). This confirms that a subtle change in acyl chain saturation is sufficient to drive lipid phase separation. Since cholesterol promotes L_o_ phase formation in vitro^53^, we tested whether rigid NE/ER membranes depend on sterol synthesis (that is, ergosterol in yeast cells). Indeed, deletion of the sterol desaturases *ERG3* or *ERG5* in Sct1-overexpressing cells reduced the apparent rigidity of NE/ER membranes (Fig. 4c,d). Thus, saturated lipids in conjunction with sterols induce rigid L_o_ phase formation in the NE/ER.

### Differential resilience of membranes to saturation stress

We then asked how other membranes respond to increased lipid saturation, focusing on the plasma membrane (PM), mitochondria and vacuoles. The PM retained its oval shape as shown by an unchanged circularity index (Extended Data Fig. 5d,e). The abundant PM H^+^-ATPase Pma1 did not undergo a detectable redistribution that would suggest a micron-scale demixing of PM lipids. Thus, the PM responds differently to increased lipid saturation compared with the NE/ER. Mitochondria are double-membrane enclosed organelles, which form a tubular reticulum in *S. cerevisiae*. TEM analysis suggested that mitochondrial tubules retained their membrane curvature and mitochondrial cristae were discernible, despite increased lipid saturation (Extended Data Fig. 5f). The mitochondrial network, visualized by Cox4–mGFP (a cytochrome c oxidase subunit), appeared more clustered (Extended Data Fig. 5g). Nevertheless, mitochondria retained their roundness, unlike the NE. Because of their irregular morphology, lipid phase separation of mitochondria is difficult to assess. To explore the possibility of lipid phase separation in a different organelle other than the NE/ER, we examined the yeast vacuole, the functional equivalent of mammalian lysosomes. The vacuolar membrane can segregate into microdomains^40,54^, resembling the demixing of synthetic membranes into L_d_ and L_o_ domains^32^. This effect is specific to stationary phase cells and was previously visualized by using Vph1, a subunit of the proton-translocating ATPase in the vacuolar membrane^40^. Upon increasing lipid saturation, we observed microdomain formation of Vph1–mScarlet during exponential growth, a condition in which vacuolar lipid phase separation is not detectable in wild-type cells^40^. Approximately 58% of cells exhibited a ‘patterned’ vacuolar distribution of Vph1 and ~7% a ‘coalesced’, half-moon distribution (Fig. 4e,f). This demonstrates that increased lipid saturation induces lipid demixing in at least one other organelle besides the NE. However, whereas the vacuole retains its spherical shape, the nucleus undergoes a massive deformation, highlighting the structural vulnerability of the NE towards saturated lipids. Hence, cellular membranes vary in their resilience to lipid saturation stress possibly due to organelle-specific lipid compositions^2^ and distinct protective mechanisms that detoxify and repair damaged membranes.

### Increased membrane lipid saturation causes NE rupture

**Fig. 5:**
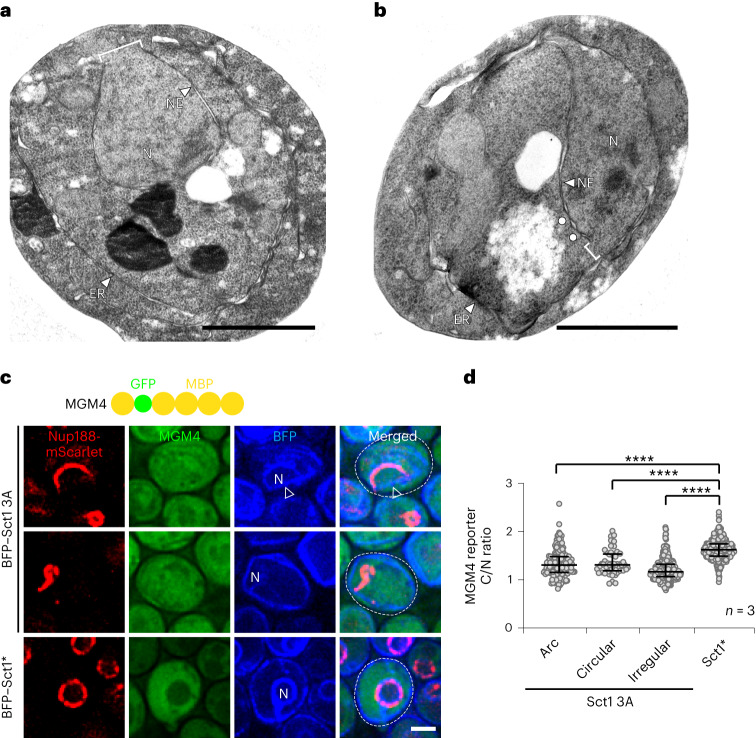
Membrane rigidification causes NE rupture. **a**,**b**, TEM examples of NE rupture upon overexpression of mGFP–*SCT1* in *sct1*Δ cells. White brackets: NE gaps (370 nm in **a**; 150 nm in **b**). White circles: pore membranes in vicinity of a rupture. N, nucleus. Scale bar, 1 µm. **c**, Nuclear leakage assay. Live imaging of *sct1*Δ cells expressing genomically tagged *NUP188*–mScarlet, genomically integrated MGM4 (MBP–GFP–4xMBP) reporter, and overexpressing plasmid-based BFP–*SCT1* constructs. Examples of ‘arc’ and ‘irregular’ NPC patterns are shown. Empty arrowhead: straight NE ‘base’. N, nucleus. Scale bar, 2 µm. **d**, Quantification of MGM4 leakage in **c**. The ratio of MGM4 fluorescence intensity in the cytoplasm and the nucleus (C/N) was calculated. NPC phenotypes were classified as ‘circular’, ‘irregular’ or ‘arc’in Sct1 3A-overexpressing cells. Median and interquartile range indicated. *P* value (****P < 0.0001) determined by two-sided Mann–Whitney test. A ratio of ‘1’ indicates breakdown of nuclear barrier function. *n*, number of biological replicates; >370 cells analysed for Sct1 3A and Sct1* cells, respectively. Source numerical data are available in source data. Source data

**Extended Data Fig. 6:**
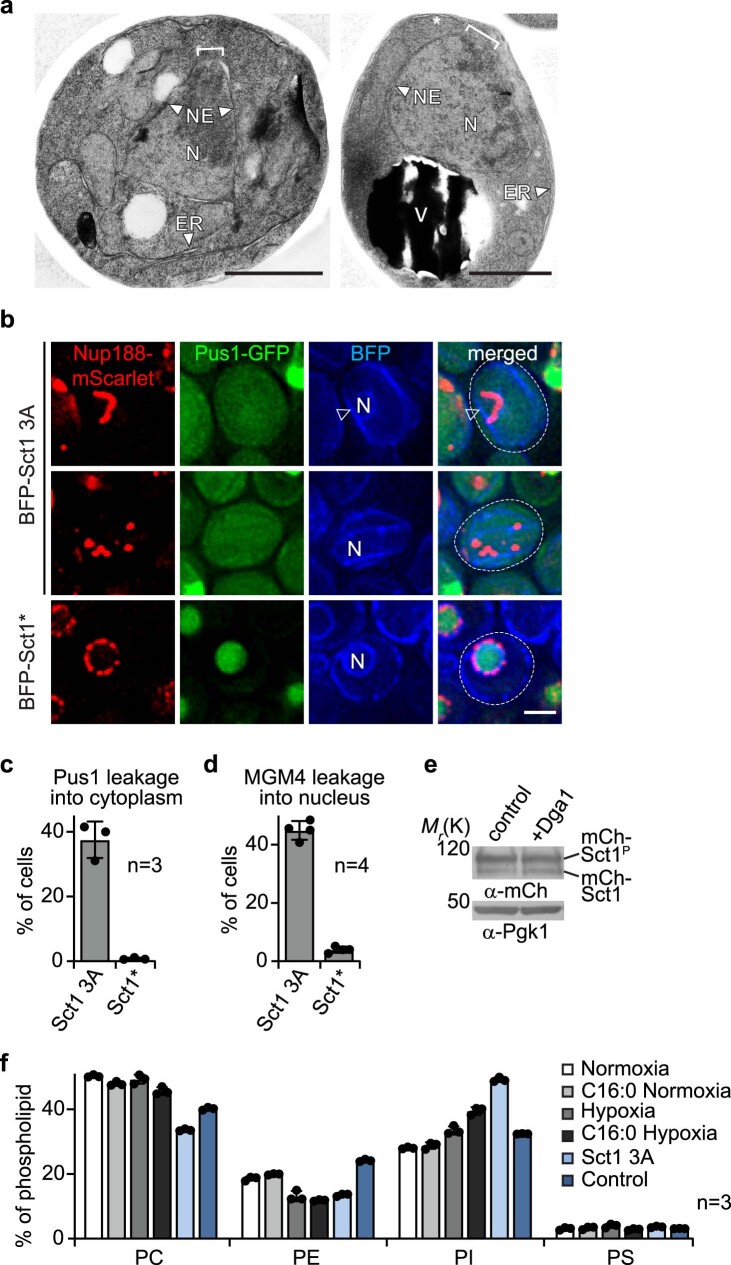
Nuclear rupture and lipidome of cells with increased lipid saturation. **a**, TEM examples of NE rupture upon overexpression of mGFP-*SCT1* from the *GAL1* promoter. White brackets mark NE gaps (240 nm in left image; 440 nm in right image). White asterisk: open flap of the torn NE. NE, nuclear envelope; N, nucleus; ER, endoplasmic reticulum; V, vacuole. Scale bar, 1 µm. **b**, Nuclear leakage assay. Live imaging of *sct1*Δ cells expressing genomically tagged *PUS1*-GFP (a tRNA:pseudouridine synthase), *NUP188*-mScarlet and overexpressing plasmid-based BFP-*SCT1* constructs. Examples of ‘arc’ and ‘irregular’ NPC patterns are shown. Empty arrowhead: straight NE ‘base’. N, nucleus. Scale bar, 2 µm. **c**, Quantification of Pus1 leakage into cytoplasm in (b). Nuclear integrity was considered normal when Pus1-GFP was enriched in the nucleus, and defective (that is leakage) when nucleoplasmic and cytoplasmic fluorescence intensities were equal. Mean value and standard deviation indicated. n = number of biological replicates; >460 cells analyzed. **d**, Quantification of nuclear leakage in (Fig. 5c). Nuclear integrity was considered normal when the MGM4 reporter was excluded from the nucleus, and as defective (that is leakage) when nucleoplasmic and cytoplasmic fluorescence intensities were equal. Mean value and standard deviation indicated. n = number of biological replicates; >450 cells analyzed. **e**, Immunoblotting for Sct1 phosphorylation. *sct1*Δ cells overexpressing plasmid-based mCherry-*SCT1* and/or mGFP*-DGA1*. Samples taken from cell cultures used in (Fig. 6b) and analyzed on a 3–8% Tris-acetate gel in Tris-acetate SDS buffer. Both constructs were overexpressed from the *GAL1* promoter. Note that Dga1 co-expression did not noticeably affect Sct1 protein levels. Upper band: phosphorylated form; lower band: unphosphorylated form. Pgk1 (3-phosphoglycerate kinase) as loading control. **f**, Lipidomic analysis. Relative abundance of four major phospholipid species (PC, PE, PI and PS). Cells supplemented with palmitic acid (C16:0) were grown under normoxia (air) or hypoxia (N_2_). Sct1 3A and control (wild-type) cells were grown for 4 h in galactose-containing media. Mean value and standard deviation indicated. n = number of biological replicates. Source numerical data and unprocessed blots are available in source data. Source data

NE membrane heterogeneities resulting from lipid phase separation may structurally destabilize the NE. *S. cerevisiae* has a closed mitosis and should not display NE discontinuities at any cell cycle stage. By TEM, however, we observed gaps in the NE, up to ~400 nm in width, indicating membrane rupture (Fig. 5a,b and Extended Data Fig. 6a). Consistent with nuclear rupture, ~37% of cells with rigid NE/ER membranes exhibited leakage of the nucleoplasmic protein Pus1 into the cytoplasm (Extended Data Fig. 6b,c). As an orthogonal approach, we analysed the localization of MGM4, a synthetic 230 kDa cytoplasmic reporter (MBP–GFP–4xMBP^55^), which is normally excluded from the nucleus. Increased lipid saturation led to nucleoplasmic localization of the reporter in ~45% of cells, again consistent with a ruptured NE (Fig. 5c and Extended Data Fig. 6d). While NE breakdown was increased in cells with ‘arc’ and ‘circular’ NPC phenotypes, it was most pronounced in cells with ‘irregular’ NPCs (Fig. 5d), indicating that this phenotype carried the greatest risk for NE breakdown. In sum, increased lipid saturation induces an aberrant demixing of NE/ER lipids and a susceptibility to nuclear rupture.

### Lipid droplets preserve NPC and NE integrity

**Fig. 6:**
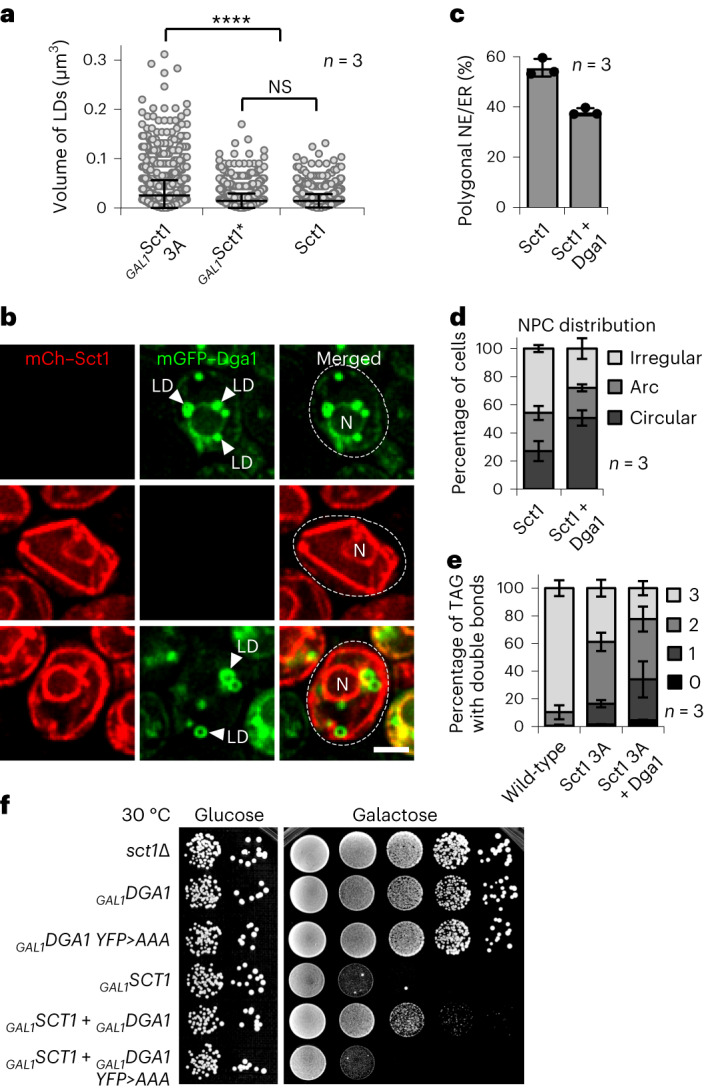
LDs sequester saturated lipids to maintain NE integrity. **a**, Automated quantification of LD volume. *n*, number of biological replicates; >1,800 cells analysed for each condition. *P* value (****P < 0.0001) determined by two-sided Mann–Whitney test. Mean value and standard deviation indicated. Sct1*, Sct1 G253L. NS, not significant (*P* = 0.708). **b**, Live imaging of *sct1*Δ cells expressing plasmid-based mCherry–*SCT1* and/or mGFP*–DGA1*. Both constructs were overexpressed from a *GAL1* promoter. White arrowheads: LDs. N, nucleus. Scale bar, 2 µm. **c**, Quantification of prevalence of polygonal NE/ER in **b**. Mean value and standard deviation indicated. *n*, number of biological replicates; >800 cells analysed for each condition. **d**, Quantification of NPC distribution in *sct1*Δ cells expressing genomically tagged *NUP188*–mScarlet and overexpressing plasmid-based *SCT1* and *DGA1*. Phenotypes classified as ‘circular’, ‘irregular’ or ‘arc’. Mean value and standard deviation indicated. *n*, number of biological replicates; >300 cells analysed for each condition. **e**, Analysis of TAG fatty acid saturation levels in LDs purified from the indicated strains. *n*, number of biological replicates. Mean value and standard deviation indicated. TAG contains three fatty acyl chains; hence, number of double bonds can range from 0 to 3. **f**, Growth of *sct1*Δ cells transformed with indicated plasmid-based constructs. *SCT1* and *DGA1* were overexpressed from a *GAL1* promoter. A catalytically inactive mutant of Dga1 (YFP motif mutated to AAA^77^) did not rescue the growth defect of Sct1 overexpression. Empty vector was used as control. Growth on plates containing glucose (repressed) or galactose (induced). Source numerical data are available in source data. Source data

Our experiments suggest that cells need to avoid excess saturated lipids to maintain NPC and NE/ER homeostasis. We have previously observed that increased lipid unsaturation, induced by Ole1 activation, increases lipid storage metabolism^46^. Interestingly, increased Sct1 activity, but not catalytically inactive Sct1, induced lipid droplet (LD) production (Fig. 6a), suggesting that increased lipid saturation also increases lipid storage metabolism. We therefore asked whether a further increase in LD production could relieve Sct1 toxicity. We overexpressed the LD-associated triacylglycerol (TAG) synthesizing enzyme Dga1 from a *GAL1* promoter in Sct1-overproducing cells. Notably, LD induction reduced the fraction of cells with a polygonal NE/ER (Fig. 6b,c and Extended Data Fig. 6e), partially restored a normal, circular NPC distribution (Fig. 6d), and increased cell viability (Fig. 6f). Lipidomic analyses confirmed that more saturated TAG was packaged into LDs in Sct1 overexpressing cells compared with wild-type cells (Fig. 6e). The amount of saturated TAG in LDs was further increased by Dga1 overexpression (Fig. 6e), explaining why more LDs are beneficial. LD biogenesis, which proceeds through highly curved membrane intermediates, may itself be restricted by rigidified membranes, limiting the buffering ability. Thus, both LDs and Ole1 can lower saturated membrane lipids to preserve NPC and NE integrity.

### Synergy of hypoxia and nutrients increase risk of NE rupture

**Fig. 7:**
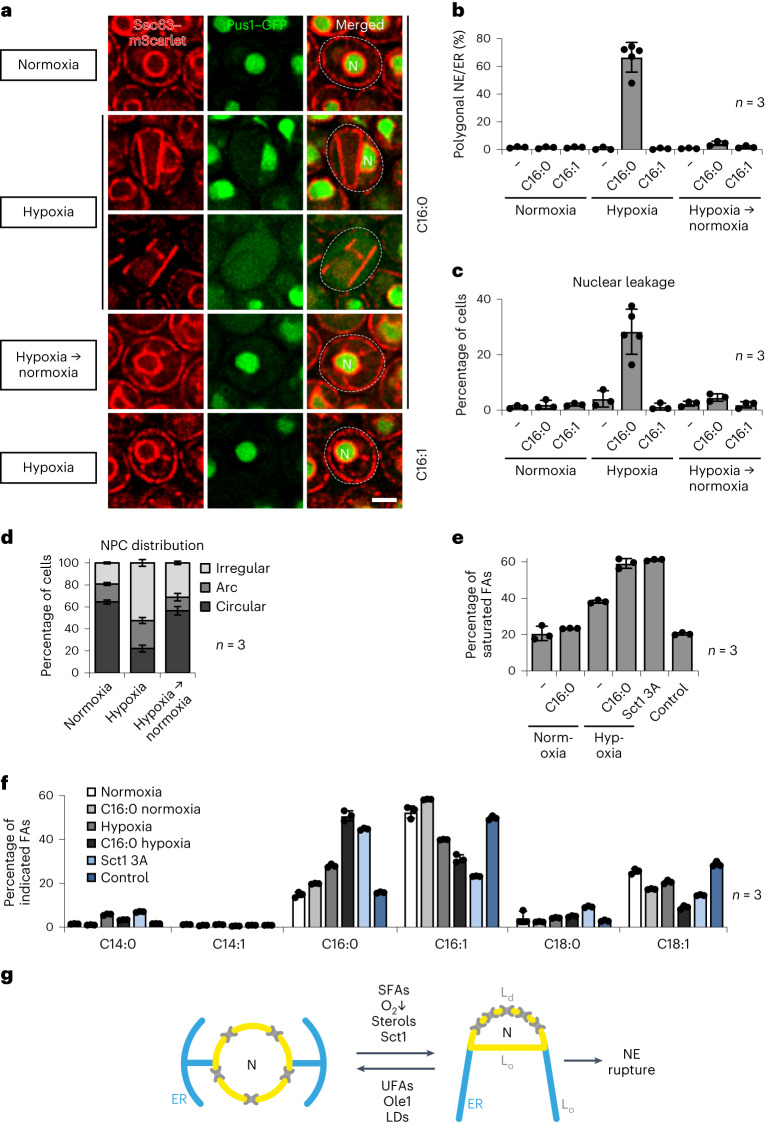
Synergistic effects of hypoxia and nutrients increase risk of NE rupture in wild-type cells. **a**, Live imaging of cells expressing genomically tagged *PUS1*–GFP and *SEC63*–mScarlet supplemented with palmitic acid (C16:0) or palmitoleic acid (C16:1). Cells were grown under normoxia (air) or hypoxia (N_2_). N, nucleus. Scale bar, 2 µm. **b**, Prevalence of polygonal NE/ER in **a**. Mean value and standard deviation indicated. *n*, number of biological replicates; >450 cells analysed for each condition. **c**, Quantification of nuclear leakage in **a**. Nuclear integrity was considered normal when Pus1–GFP was enriched in the nucleus, and defective (that is, leakage) when nucleoplasmic and cytoplasmic fluorescence intensities were equal. Mean value and standard deviation indicated. n, number of biological replicates; >450 cells analysed for each condition. **d**, Quantification of NPC distribution in cells expressing genomically tagged *NUP188*–GFP and *SEC63*–mScarlet. Cells were grown under normoxia (air) or hypoxia (N_2_), both with palmitic acid (C16:0) supplementation. NPC phenotypes classified as ‘circular’, ‘irregular’ or ‘arc’-like as in Fig. 3a. Mean value and standard deviation indicated. *n*, number of biological replicates; >650 cells analysed for each condition. **e**, Lipidomic analysis of SFA content in the indicated samples, showing a composite of major FA species, as indicated in **f**. Mean value and standard deviation indicated. *n*, number of biological replicates. **f**, Breakdown of the FA species shown in **e**. Cells were grown under normoxia (air) or hypoxia (N_2_). Sct1 3A and control cells (wild type) were grown for 4 h in galactose-containing medium. Mean value and standard deviation indicated. *n*, number of biological replicates. **g**, Model of lipid-saturation-induced transition of the NE/ER into rigid L_o_ domains and more elastic L_d_ domains with a concomitant segregation of NPCs. Factors that were identified to catalyse this reversible transition are listed. NE rupture reflects a failure to resolve lipid saturation imbalances. N, nucleus. Source numerical data are available in source data. Source data

By genetically increasing the levels of endogenously synthesized saturated lipids, we uncovered their role in NE rigidification and NE rupture. To address whether this mechanism is physiologically relevant in wild-type cells, we exposed them to exogenous SFAs (palmitic acid; C16:0). Wild-type cells expressing Sec63–mScarlet as an NE/ER marker and Pus1–GFP as a nucleoplasmic marker showed no signs of NE/ER rigidification (Fig. 7a,b). Thus, SFA overload is sufficiently buffered by Ole1’s desaturase activity. Ole1 requires a mini-redox system^24,56,57^. To introduce a double bond into a saturated acyl chain, electrons must transfer from NADH to molecular oxygen, which renders the reaction oxygen dependent. Hence, we tested the combined effect of oxygen deprivation (hypoxia; that is, replacing ambient air with N_2_ during cell growth) and SFAs (C16:0). Strikingly, under this condition ~66% of wild-type cells exhibited a rigidified NE/ER, the same phenotype seen upon Sct1 overexpression under normoxia (Fig. 7a,b). Moreover, ~30% of wild-type cells in the hypoxic condition exhibited leakage of Pus1, indicating NE rupture (Fig. 7c). Membrane rigidification was reversible upon reoxygenation (Fig. 7a,b). Leakage of Pus1 was also reduced upon reoxygenation (Fig. 7c), suggesting NE repair^58^. Rigidification and rupture of the NE were specific to the combination of hypoxia and SFAs (C16:0) and were not seen in hypoxia alone or when combining hypoxia and UFAs (C16:1). Combining hypoxia and SFAs also caused a large-scale segregation of NPCs into NPC ‘arcs’ or ‘irregular’ NPC patterns (Fig. 7d), similar to the effects of Sct1 overexpression. The NPC phenotypes were also largely reversible upon reoxygenation. Finally, we asked whether hypoxia and Sct1 overexpression elicit NE/ER rigidification through a similar lipidomic fingerprint. We determined the cellular abundance of saturated FAs (C14:0, C16:0 and C18:0) (Fig. 7e). Notably, SFAs increased to ~60% in cells overexpressing Sct1 3A as well as in hypoxic cells grown with palmitic acid (C16:0). In comparison, normoxic cells contained only ~20% SFAs and these levels were largely unchanged by C16:0 addition, probably because Ole1 is active. Hypoxia alone had an intermediate effect, raising SFA levels to ~38%. The lipidomic adaptations to hypoxia and Sct1 overexpression were mostly driven by an increase in C16:0 and decrease in C16:1 and C18:1 (Fig. 7f), consistent with an earlier analysis of the Sct1 phenotype^27^. Of note, both hypoxia with SFA supplementation and Sct1 3A overexpression lower the phosphatidylethanolamine (PE) content of cells, while increasing phosphatidylinositol (PI) levels (Extended Data Fig. 6f). These alterations may counter the effect of saturated acyl chains, as the smaller PE head group allows tighter packing of acyl chains, and hence rigidification of membranes^25,27,59^. Consistently, increased PI levels suppress the growth defect of cells with increased lipid saturation, explaining why increased PI levels could be beneficial under hypoxic conditions or Sct1 overexpression^60^. Taken together, our lipidomic analyses support the notion that NE/ER rigidification is caused by increased lipid saturation and that a genetically engineered increase of acyl chain saturation is mechanistically similar to environmentally induced lipid imbalances in wild-type cells.

## Discussion

In this study, we have gained important insights into the relevance of lipid unsaturation homeostasis, the factors involved and the threshold at which a functional NE breaks down (Fig. 7g). We describe how cells behave at this transition: they develop micron-scale lipid phase separation of the NE/ER into rigid L_o_ domains and more elastic L_d_ domains with aberrant segregation of NPCs into the latter. Cell nuclei surrounded by such membranes become susceptible to rupture, causing nuclear leakage and exposure of chromosomal DNA to the cytoplasm. Aspects of the structural remodelling of the cell nucleus, including the development of planar membranes and the occurrence of lipid phase separation, can be recapitulated in vitro.

Along with NE geometry, NPC architecture, distribution and function depend on lipid unsaturation homeostasis. A saturated lipid acyl chain profile impairs the incorporation of lipid-interacting Nups and the formation of a properly curved pore membrane. NPCs strongly prefer the L_d_ phase with an enrichment of unsaturated lipids over the L_o_ phase. This may indicate that NPCs are naturally surrounded by an elastic ‘belt’ of disordered, malleable lipids, endowing them with conformational plasticity and responsiveness to membrane tension^6,10,15^. During NPC biogenesis, lipid disorder could favour pore curvature formation. Conversely, a curved membrane may attract unsaturated lipids. Indeed, curvature-induced lipid sorting was previously observed in synthetic membranes^35^. Lipid phase separation promotes membrane fission in vitro^35^, which could be harnessed locally to catalyse the fusion/fission of INM and ONM during interphase NPC biogenesis^5,61,62^.

Our results further show that different organelles display varying resilience to lipid saturation stress. The vulnerability of the NE/ER can be attributed to several factors: as the primary site of lipid biosynthesis, the ER undergoes changes more directly than other organelles and its lipids readily reach the NE by NE/ER junctions. Other membrane compartments may be protected by the number or nature of their membrane contact sites and the routes of vesicular trafficking. Furthermore, other membranes, such as the PM, possess a higher proportion of tightly packed saturated lipids compared with the NE/ER^1,63^, potentially making them more resilient. Finally, the unique lipid compositions specific to each organelle (for example, sterol content) may impact their propensity for lipid phase separation.

The NE is susceptible to rupture when lipid saturation increases, which can have grave consequences. We suggest that the demixing of NE lipids creates fracture-prone transition zones. In vitro, lipid bilayers in cholesterol-rich L_o_ phases are thicker than those in cholesterol-poor L_d_ phases, leading to a height mismatch at the phase boundaries^64^. An interfacial energy called ‘line tension’ builds up at phase boundaries, which could trigger NE rupture in vivo. Importantly, rigidified and fracture-prone nuclei are not only observed upon genetically rewiring cellular lipid fluxes. The nuclei of wild-type cells react similarly when exposed to nutrient SFAs and hypoxia. A combination of hypoxia and fatty acid overload may be encountered in natural habitats of *S. cerevisiae*, which is a facultative anaerobe^65–67^. Hypoxia is also a frequent condition in human tumours^68^, resulting from high cell proliferation rates and insufficient blood supplies. Both budding yeast and human cancer cells respond to oxygen deprivation by upregulating the *OLE1*/*SCD1* desaturase, probably to offset the reduced activity of these enzymes under hypoxia^69–71^. Additionally, both yeast and cancer cells increase uptake of exogenous lipids^72–74^. Thus, the ability to adapt to changes in environmental oxygen and lipid saturation may be governed by evolutionary conserved mechanisms. NE membranes of hypoxic cancer cells may be particularly vulnerable to lipid saturation imbalances and prone to NE rupture, as seen in yeast. Although NE rupture can promote cancer progression by increasing DNA damage, aneuploidy and genomic rearrangements^75^, it may also present a specific weakness of cancer cells that could be targeted by drugs that alter the lipid saturation balance of the cell nucleus.

## Methods

### Strains and media

Yeast strains and plasmids used in this study are listed in Supplementary Tables 1 and 2, respectively. Genes were tagged by a standard one-step PCR-based technique. Microbiological techniques followed standard procedures. To induce expression from the *GAL1* promoter, cells were grown in media containing 2% raffinose before adding galactose to a final concentration of 2% for 4 h, except for Figs. 1e and 2a and Extended Data Figs. 1d and 2a where induction was carried out for 8 h, and Fig. 4e for 6 h and Fig. 6e for 5 h.

For LA treatment, cells were grown for 2 h in galactose medium to induce Sct1, before addition of 0.5% LA and 1% Tween 80 (both Sigma-Aldrich).

For α-factor treatment, cells were grown for 2 h in SRC medium before α-factor addition (10 µg ml^−1^) and grown for an additional 2.5 h. Galactose (2% final) was then added to induce Sct1 expression. After 30 min, cells were washed three times with SRC + 2% galactose to remove α-factor, and grown for another 3 h before imaging.

For the RITE, the overnight yeast culture was supplemented with 250 µg ml^−1^ hygromycin. After dilution in galactose-containing medium, 250 µg ml^−1^ hygromycin or 2 µm estradiol was added and cells were grown for 4 h before imaging.

To induce hypoxia, cultures were exposed to a continuous flow of N_2_ (~3.5 l min^−1^) in a closed system for 6 h. The experimental set-up was adapted from ref. ^78^. Normoxic controls were grown for 6 h under ambient air before analysis. For re-oxygenation experiments, hypoxic cultures (6 h growth) were grown for an additional 4 h under normoxia. Fatty-acid-containing growth media were prepared from standard synthetic dextrose complete (SDC) media containing 0.1% glucose, 16 mM palmitic or palmitoleic acid (both from Sigma-Aldrich) and 2.5% Brij-35 (30% solution, Fisher Scientific or Sigma-Aldrich). Control media contained 2.5% Brij-35 without fatty acids. Media for lipidomic experiments were filtered before use with a 5 µM filter (syringe filter CHROMAFIL Xtra PES, 5.0 µm, Carl Roth).

### Live-cell imaging of yeast

Exponentially growing cells were immobilized on microscope slides with agarose pads and imaged on a DeltaVision Elite microscope (GE Healthcare). Images were acquired with a 60× oil immersion objective and recorded with a CoolSNAP HQ2 CCD camera (Photometrics). Deconvolution was carried out using softWoRx software (GE Healthcare). Images were processed with ImageJ. Cell contours were marked with a dashed white line based on brightfield imaging. To stain LDs or vacuoles, BODIPY 493/503 (final concentration 5.7 μM) or CellTracker Blue (final concentration 10 μM) were added, respectively, and cells were imaged after 20 min. For Laurdan imaging, cells were stained with 5 µM Laurdan (stock 1 mg ml^−1^ in dimethyl sulfoxide, Thermo Fisher Scientific) for 1 h. Images were recorded on an SP8 DIVE confocal (inverted) microscope with multi-photon lasers (Leica) equipped with a confocal detector HyD (350–800 nm) and 4 Tune Detector HyD NDD (380–800 nm), using a 63×/1.30 glycerol objective. Laurdan was excited at 807 nm, and detection windows were set to 400–460 nm and 470–516 nm.

### Preparation and imaging of GUVs

Lipids DOPC (18:1 (Δ9-Cis) PC, 1,2-dioleoyl-*sn*-glycero-3-phosphocholine), 16:1 PC ((Δ9-Trans) PC, 1,2-dipalmitelaidoyl-*sn*-glycero-3-phosphocholine), DPPC (16:0 PC, 1,2-dipalmitoyl-*sn*-glycero-3-phosphocholine), Rhod-PE (18:1 Liss Rhod-PE, 1,2-dioleoyl-*sn*-glycero-3-phosphoethanolamine-*N*-(lissamine rhodamine B sulfonyl) (ammonium salt)) and cholesterol (ovine wool, 98%) were obtained from Sigma-Aldrich. Two different lipid mixes were prepared (Supplementary Table 3). GUVs were stained with Laurdan at 0.5 mol% and Rhod-PE at 0.1 mol%. GUVs were synthesized by polyvinyl alcohol (PVA)-assisted swelling^79^. One-hundred microlitres of 5% (w/w) PVA solution (weight-average molecular weight of 146,000 to 186,000, Sigma-Aldrich) was spread on a glass coverslip and dried at 60 °C for 40 min. Twenty microlitres of lipid mixture (1 mg ml^−1^) in chloroform was spread on top of the PVA layer and chloroform was allowed to evaporate for 1 h. Five-hundred microlitres of swelling buffer (280 mM sucrose and 10 mM Tris (pH 7.5)) was added on the coverslip and incubated for 1 h at room temperature to induce vesicle formation. Vesicles were collected by pipetting and kept at room temperature for immediate use. Vesicles were imaged in 16-well plates (glass bottom, Grace BioLabs). Wells were blocked for 1 h with coating buffer (100 mM NaCl, 50 mM Tris (pH 7.5) and 2.5 mg ml^−1^ bovine serum albumin). Coated wells were washed with buffer (100 mM NaCl, 50 mM Tris (pH 7.5) and 1.5 mM MgCl_2_) before loading the samples. Ten microlitres of the GUV solution was mixed with 50 µl buffer (100 mM NaCl, 50 mM Tris (pH 7.5) and 1.5 mM MgCl_2_), and 50 µl was loaded in the well for imaging. Laurdan images were recorded on an SP8 DIVE confocal (inverted) microscope with multi-photon lasers (Leica) as described above.

### GUV reconstitution from whole cell lipid extracts

Sixty optical densities (ODs) of Sct1 3A or Sct1* cells grown for 4 h in galactose-containing medium were collected, washed twice with water and frozen in N_2_. After de-freezing, cells were resuspended in 150 µl water, 200 µl glass beads (0.4–0.6 mm) were added and samples were vortexed for 30 min at 4 °C. Lysates were transferred to 2 ml tubes, and water was added to 200 µl. One millilitre of chloroform/methanol (1:1) was added and samples mixed by vortexing, before adding 500 µl chloroform and 300 µl water and mixing again by vortexing. Tubes were centrifuged at room temperature for 5 min at 12,300*g*. After centrifugation, an aqueous top phase and organic bottom phase, which contains the lipids, were obtained. The lipid phase was collected and the solvent evaporated at room temperature using an Eppendorf Concentrator Plus. The lipid film was diluted in 100 µl chloroform and 1 µl Rhod–PE (0.1 mg ml^−1^) was added. GUVs were prepared as described in ‘Preparation and imaging of GUVs’. GUVs were imaged in 16-well plates (glass bottom, Grace BioLabs) using a DeltaVision Elite microscope (GE Healthcare). Images were acquired with a 60× oil immersion objective and recorded with a CoolSNAP HQ2 CCD camera (Photometrics). Deconvolution was carried out using softWoRx software (GE Healthcare).

### Yeast growth assay

For dot-spot assays, cells were grown exponentially in SRC drop-out medium, collected and resuspended to a final OD_600_ of 0.5. Ten-fold serial dilutions were prepared, spotted on appropriate plates and incubated at 30 °C. Where indicated, plates contained 2% galactose and 0.5% LA and 1% Tween 80 (both Sigma-Aldrich).

### TEM

Wild-type cells were grown in SDC medium. *sct1*Δ cells expressing mGFP–*SCT1* constructs were grown for 4 h in SRC medium supplemented with 2% galactose. Pelleted cells were mixed 1:1 with 10% bovine serum albumin, used as a filler and transferred into the 100 µm cavity of a 3 mm aluminium specimen carrier. This carrier was sandwiched with a flat 3 mm aluminium carrier and immediately high pressure frozen in an HPF Compact 01 (both carriers and high-pressure freezer from Engineering Office M. Wohlwend GmbH). The frozen samples were subsequently transferred into a Leica EM AFS-2 freeze substitution unit (Leica Microsystems). Over a period of 4 days, samples were substituted in a medium of acetone containing 2% osmium tetroxide (Agar Scientific), 0.2% uranyl acetate and 5% water. Freeze substitution was performed according to the following protocol: 40 h at –90 °C, warm up at a rate of 2 °C per hour to −54 °C, 8 h at −54 °C, warm up at a rate of 5 °C per hour to −24 °C, 15 h at −24 °C, warm up at a rate of 5 °C per hour to 0 °C, 2 h at 0 °C. At 0 °C, samples were taken out and washed three times in anhydrous acetone (on ice) and infiltrated with Agar 100 Epoxy resin (Agar Scientific) in a graded series of acetone and resin over a period of 3 days. Polymerization took place at 60 °C. Ultrathin sections with a nominal thickness of 70 nm were cut using a Leica UCT ultramicrotome (Leica Microsystems). Regions on the sections were randomly selected and inspected with a FEI Morgagni 268D (FEI) operated at 80 kV. Digital images were acquired using an 11 megapixel Morada CCD camera (Olympus-SIS).

### Electron tomography

For electron tomography, cells were prepared as described in the previous section, except that 150–250 nm sections were cut on a Leica UCT ultramicrotome (Leica Microsystems). After collecting the sections on a 50 mesh Cu/Pd grid (Gilder Grids), previously coated with a supporting film of formvar, the section was covered on both sides with 10 nm gold (Aurion) by incubating the grid in a drop of concentrated gold solution for 3 min. Baking for 30 min was performed before collecting the tilt series to avoid shrinkage of the sample. Tilt series were acquired at a Tecnai G2 20 microscope (FEI) equipped with an Eagle 4k HS CCD camera (FEI) and operated at 200 kV. Dual axis tilt series were collected with a maximum tilting range from −60° to +64° at 1° increments. For data acquisition, processing and modelling, the IMOD software^80^ from the Boulder Laboratory for 3D Electron Microscopy of Cells was used.

### Correlative light and electron microscopy

*sct1*Δ cells expressing plasmid-based *sct1 3A* under the *GAL1* promoter and genomically tagged *NUP188*–GFP were grown in medium containing 2% galactose for 4 h. Cells were filtered via Whatman Nucleopore filter membranes (Merck) with 0.1 µm pore size. The filtered cells were filled into the 100 µm cavity of an aluminium platelet (3 mm in diameter) covered with a flat carrier and immediately high pressure frozen in an HPF compact 01 (both high pressure freezer and platelets from Engineering Office M. Wohlwend GmbH). The frozen samples were transferred into an automated freeze substitution unit, a Leica EM AFS-2 (Leica Microsystems). Over a period of 3 days, samples were substituted with a medium of acetone containing 0.1% uranyl acetate (Merck). Freeze substitution was performed as follows: 48 h at −90 °C, warm up at a rate of 3 °C per hour to −54 °C, 8 h at −54 °C, warm up at a rate of 3 °C per hour to −25 °C, 10 h at −25 °C. At a temperature of –25 °C samples were washed in acetone and infiltrated with freshly prepared HM20 Lowicryl resin (EMS) using graded series of resin. Polymerization of the pure resin took place at −40 °C under ultraviolet light for 12 h. Subsequently the polymerized resin was warmed up to room temperature and kept in the dark for at least 48 h before trimming and sectioning.

The HM20 resin blocks were trimmed to a pyramid (Leica EM Trim) and sectioned with a Leica UCT ultramicrotome (both Leica Microsystems). For fluorescence microscopy, one section of 250 nm (nominal thickness) was cut and mounted on a glass slide using ProLong Glass Antifade (Sigma-Aldrich), followed by up to three thin sections (70 nm nominal thickness) for electron microscopy collected on Cu/Pd slot grids (Agar Scientific), coated with a supporting film of formvar.

Fluorescence imaging was performed using an inverted Zeiss Axio Observer 7 (Carl Zeiss AG) Fluorescence Microscope with a plan-apochromat 63×/1.4 oil DIC RMS objective and an Orca Flash 4.0 V3 camera (Hamamatsu Photonics). *Z*-stacks of fluorescence and DIC images were acquired with ZEISS ZEN blue 3.1 pro software (Carl Zeiss AG). Images were processed with ImageJ.

For electron micrsocopy, images were recorded in a FEI Tecnai G2 20 TEM, operated at 200 kV and equipped with an Eagle 4k HS CCD camera (both FEI). Post-processing of images was performed with ImageJ.

Using the ZEISS ZEN Blue 3.3 Desk with ZenConnect Module (Carl Zeiss AG), light and electron microscope images were manually correlated optimizing the best fit of cell contours.

### LD isolation

Yeast cultures were grown in Synthetic Galactose Complete medium for ~5 h before collecting and washing with water and Zymolyase buffer (1 M sorbitol, 50 mM Tris–HCl, (pH 7.5) and 10 mM MgCl_2_). Cell pellets were frozen in liquid N_2_ and after de-freezing 25 ml Zymolyase buffer and dithiothreitol (DTT; 3 mM final) were added. Cells were incubated for 15 min at 30 °C with shaking and centrifuged for 5 min at 3,434*g*. Cell pellets were resuspended in 18 ml Zymolase buffer and 20 µl 1 M DTT. Thirty milligrams Zymolyase 20T (AMSBIO) was added, and samples were incubated at 30 °C for 1.5 h on a turning wheel. After spheroplasting, cold 1 M sorbitol was added to adjust the volume to 40 ml, and cells were pelleted at 3,434*g* for 10 min at 4 °C. The pellet was resuspended in 40 ml 1 M cold sorbitol and cells were pelleted at 3,434*g* for 10 min at 4 °C. The pellet was resuspended on ice in 5 ml lysis buffer (8% PVP-40, 20 mM K-phosphate (pH 6.5), 7.5 µM MgCl_2_ and 25 μl 1 M DTT). Before cell lysis, 50 μl of phenylmethanesulfonyl fluoride (20 mg ml^−1^ stock) in ethanol and 50 μl protease inhibitor cocktail (PIC, Serva) were added. Spheroroplasts were homogenized using a Dounce homogenizer. An 8% PVP-40 solution containing 1:1,000 PIC was added to adjust the volume to ~18 ml. Lysate was loaded on top of a sucrose gradient (8 ml 2.50 M sucrose, 10 mM Bis–Tris–HCl (pH 6.5), 0.1 mM MgCl_2_/1.875 M sucrose, 10 mM Bis–Tris–HCl (pH 6.5), 0.1 mM MgCl_2_ containing phenylmethanesulfonyl fluoride and PIC). Gradients were centrifuged at 96,000*g* for 30 min at 4 °C. The upper LD fraction was loaded on top of the cushion buffer (20 mM HEPES/KOH (pH 7.4) and 20 mM KCl). Centrifugation was performed at 85,000*g* for 1 h at 4 °C. The upper LD fraction was removed and frozen in liquid N_2_.

### Lysate preparation for whole cell lipidome analysis

Twenty ODs of cells were grown under normoxia (air) or hypoxia (N_2_ flow) as described in ‘Strains and media’; Sct1 3A or wild-type cells were grown for 4 h in galactose-containing medium. After collecting, cells were washed three times with water and frozen in liquid N_2_. After de-freezing, cells were resuspended in 150 µl water, 200 µl glass beads (0.4–0.6 mm) were added and samples were vortexed for 30 min at 4 °C. A total of 850 µl water was added to reach a concentration of 20 OD ml^−1^. Then 700 µl was transferred to a 2 ml tube and frozen in liquid N_2._

### Mass spectrometry of lipids

Lipidomic analysis of LDs and whole cell lysates was performed by Lipotype GmbH as described^81,82^. Lipids were extracted using a two-step chloroform/methanol procedure^81^. LD samples were spiked with triacylglycerol 17:0/17:0/17:0 (TAG), whereas whole cell lipidome samples were spiked with internal lipid standard mixture containing: CDP-DAG 17:0/18:1, cardiolipin 14:0/14:0/14:0/14:0 (CL), ceramide 18:1;2/17:0 (Cer), diacylglycerol 17:0/17:0 (DAG), lyso-phosphatidate 17:0 (LPA), lyso-phosphatidylcholine 12:0 (LPC), lyso-phosphatidylethanolamine 17:1 (LPE), lyso-phosphatidylglycerol 17:1 (LPG), lyso-phosphatidylinositol 17:1 (LPI), lyso-phosphatidylserine 17:1 (LPS), phosphatidate 17:0/14:1 (PA), phosphatidylcholine 17:0/14:1 (PC), phosphatidylethanolamine 17:0/14:1 (PE), phosphatidylglycerol 17:0/14:1 (PG), phosphatidylinositol 17:0/14:1 (PI), phosphatidylserine 17:0/14:1 (PS), ergosterol ester 13:0 (EE), triacylglycerol 17:0/17:0/17:0 (TAG), stigmastatrienol, inositolphosphorylceramide 44:0;2 (IPC), mannosyl-inositolphosphorylceramide 44:0;2 (MIPC) and mannosyl-di-(inositolphosphoryl)ceramide 44:0;2 (M(IP)_2_C). After extraction, the organic phase was transferred to an infusion plate and dried in a speed vacuum concentrator. First-step dry extract was resuspended in 7.5 mM ammonium acetate in chloroform/methanol/propanol (1:2:4, V:V:V) and second-step dry extract in 33% ethanol solution of methylamine in chloroform/methanol (0.003:5:1, V:V:V). All liquid handling steps were performed using a Hamilton Robotics STARlet robotic platform with the Anti Droplet Control feature for organic solvent pipetting. Samples were analysed by direct infusion on a QExactive mass spectrometer (Thermo Scientific) equipped with a TriVersa NanoMate ion source (Advion Biosciences). Samples were analysed in both positive and negative ion modes with a resolution of *R*_*m*/*z*=200_ = 280,000 for mass spectrometry (MS) and *R*_*m*/*z*=200_ = 17,500 for tandem mass spectrometry (MS/MS) experiments, in a single acquisition. MS/MS was triggered by an inclusion list encompassing corresponding MS mass ranges scanned in 1 Da increments^83^. Both MS and MS/MS data were combined to monitor EE, DAG and TAG ions as ammonium adducts; PC as an acetate adduct; and CL, PA, PE, PG, PI and PS as deprotonated anions. MS only was used to monitor CDP-DAG, LPA, LPE, LPG, LPI, LPS, IPC, MIPC and M(IP)_2_C as deprotonated anions; Cer and LPC as acetate adducts and ergosterol as protonated ion of an acetylated derivative^84^. Data were analysed with a lipid identification software based on LipotypeXplorer^85,86^. For data post-processing, only lipid identifications with an S/N ratio >5, and a signal intensity five-fold higher than in corresponding blank samples were considered for further data analysis.

### Immunoblotting

Yeast whole-cell extracts were prepared, normalized for protein concentration and analysed by immunoblotting according to standard procedures. Antibodies were used according to the manufacturer’s instructions: mouse monoclonal anti-mCherry (clone 1C51) (1:2,000, Abcam cat. no. ab125096), mouse monoclonal anti-GFP (clones 7.1 and 13.1) (1:2,000, Roche cat. no. ab11814460001), mouse monoclonal anti-Pgk1 (clone 22C5D8) (1:10,000, Abcam cat. no. ab113687), peroxidase AffiniPure Goat anti-Mouse IgG (polyclonal) (1:5,000, Jackson ImmunoResearch cat. no. 115035008) and mouse monoclonal anti-HA (clone 12CA5) (1:1,000, Max Perutz Labs Monoclonal Antibody Facility).

### Statistics and reproducibility

Number of biological replicates is indicated in the figures, and sample size in the figure legends. All microscopy experiments were repeated at least three times, except Figs. 2a,b and 4b and Extended Data Fig. 3p, which were repeated two times. Immunoblotting experiments were repeated two times, except Extended Figs. 2b and 6e, which were repeated five times. Yeast growth assays were repeated at least two times. Lipidomic experiments were repeated three times. EM-based experiments were done once. All attempts to replicate the data were successful. Data normality was determined using the Shapiro–Wilk test. Statistical significance was evaluated by two-tailed *t*-test or Mann–Whitney test depending on data normality using the GraphPad Prism software, where indicated. Statistical significance is indicated in figures (^∗^*P* < 0.05, ^∗∗^*P* < 0.01, ^∗∗∗^*P* < 0.001, ^∗∗∗∗^*P* < 0.0001). No statistical method was used to pre-determine sample size. No data were excluded from the analyses, unless mentioned in the description of the analysis below. The experiments were not randomized. The investigators were not blinded to allocation during experiments and outcome assessment. Detailed descriptions of phenotype quantifications are provided below:

- The NE/ER phenotype was classified as polygonal if at least two ER membranes intersected at an angle less than 180°.
- To quantify NPC defects, we analysed how many cells with polygonal ER membranes exhibit a ‘circular’, ‘irregular’ or ‘arc’ distribution of NPCs (‘arc’ defined as NPCs occupying ~30–60% of the NE). The frequency of ectopic NPC foci was quantified only in cells with ‘arc’ or ‘circular’ NPC patterns. An ‘ectopic’ NPC focus must be located outside of an NPC ‘arc’ or NPC circle. Ectopic NPCs were further quantified with respect to Nup co-localization. To quantify the reversal of NPC defects upon Dga1 overexpression or LA addition, every cell that contained Sct1 fluorescence was analysed.
- To quantify mitotic spindle phenotypes, only dividing cells that exhibited similarly sized mother and daughter cells were considered (anaphase). A spindle was considered as ‘regular’ if nuclear microtubules (MTs) were longer than cytoplasmic MTs, aligned with the cell division axis, and cytoplasmic MTs were short. A spindle was considered ‘irregular’ if nuclear MTs were misaligned, discontinuous or branched, and cytoplasmic MTs were long or extensively branched.
- To quantify SPB co-segregation with NPCs, only cells exhibiting NPC ‘arcs’ and polygonal ER membranes were analysed. Only SPBs in the same plane of focus with NPC ‘arcs’ were scored for co-segregation with NPCs. If co-segregated, the position of SPBs relative to the NPC ‘arc’ was determined by dividing the ‘arc’ in four equal sections. SPB localization in the outer 25% of the ‘arc’ (one or two SPBs) was considered as peripheral.
- To quantify nuclear import efficiency with the NLS–2xmCherry reporter, the mean signal intensity of the nucleus (N) was measured using ImageJ. An identically sized area was used for measuring the mean signal intensity of the cytoplasm (C). The N/C ratio of the NLS–2xmCherry fluorescence intensities was then calculated. Before statistical analysis, outliers were removed using the ROUT method (*Q* set to 1%) in the GraphPad Prism software.
- To quantify incorporation of new NPCs into the NE after RITE, we scored whether the Nup188-GFP signal (‘new’ NPCs) co-segregated with the Nup188–mCherry signal (‘old’ NPCs). Only cells containing NPC ‘arcs’ were analysed.
- To determine INM LipSat sensor localization, an identically sized area of the NE and nucleoplasm were selected in ImageJ and signal intensities were measured before calculating the LipSat NE/nucleoplasm ratio.
- For the analysis of INM LipSat sensor processing, the sum of the total NLS–Mga2 protein was calculated from the immunoblots, and the percentage of NLS–p120* and NLS–p90* relative to the total amount was determined. The intensity of the bands was measured in ImageJ.
- To quantify the circularity of cellular structures (that is, H2B for the nucleus and Pma1 for the PM), the contours were marked in ImageJ and the circularity index was determined.
- To quantify nuclear rupture and leakage, nuclear integrity was considered normal if the Pus1–GFP signal was enriched in the nucleus. Pus1 leakage was defined as equal fluorescence intensities of Pus1–GFP in the nucleoplasm and the cytoplasm.
- To quantify nuclear leakage with the MGM4 reporter, nuclear integrity was considered normal if the MGM4 reporter was excluded from the nucleus. MGM4 leakage into the nucleus was defined as equal fluorescence intensities of MGM4 in the nucleoplasm and the cytoplasm. To quantify the ratio of MGM4 reporter, the mean signal intensity of the nucleus (N) was measured using ImageJ. An identically sized area was used for measuring the mean signal intensity of the cytoplasm (C). The C/N ratio of the MGM4 fluorescence intensities was then calculated.
- To quantify vacuolar lipid phase separation, we analysed how many cells with polygonal NE/ER membranes exhibited vacuole microdomains in cross-section. ‘Patterned’ was defined as multiple small domains of Vph1–mScarlet with gaps in between. ‘Coalesced’ was defined as a single Vph1–mScarlet domain (that is half-moon) within the vacuole. A homogeneous Vph1–mScarlet signal was classified as ‘None’. If vacuoles were too small or Vph1 staining too faint for reliable quantification, the phenotype was classified as not determinable ‘n.d.’.
- Automated quantification of cellular LD volume was performed using the Fiji plugin ‘Trainable Weka Segmentation’. The segmentation classifier was trained with two classes: to recognize LDs (class 1) and to recognize the background (class 2). The ‘Watershed’ plugin was used to separate adjacent LDs. Next, particle analysis was performed to obtain the area of each LD, and the volume was calculated assuming that each LD is spherical. Two extreme outliers (out of 4,477 data points) were excluded from the analysis.
- For analysis of TAG double bonds, the threshold for TAG lipid species was set ≥0.5 mol%. The sum of all TAG lipid species after thresholding was set to 100% of TAG.
- The relative abundance of four major phospholipid species (PC, PE, PI and PS) in the whole cell lipidome was calculated by setting the pmol sum of these lipids to 100%.
- Analysis of GUVs reconstituted from whole cell lipid extracts was based on Rhod–PE signal. If the Rhod–PE distribution was not homogeneous, the GUV was classified as ‘phase-separated’. Phase-separated GUV that exhibited edges were classified as ‘angular’. Fused GUVs were excluded from the analysis.
- Laurdan data are displayed as pseudocoloured generalized polarization (GP) images. The calculation of the GP images was performed in Fiji as described in^52^ using the provided custom-written macro. GP is calculated according to the following equation:
  $${{\mathrm{GP}}}=\frac{{I}_{400-460}-{{{GI}}}_{470-516}}{{I}_{400-460}+{{{GI}}}_{470-516}}$$
  where *I* represents the intensity in each pixel in the image acquired in the indicated spectral channel (numbers are in nm) and *G* is the calibration factor. *G* factor was set to 1.
- GP values for a region of interest were determined by a custom-written macro^52^. After selecting a region in Fiji, the histogram function provides mean intensity values and pixel counts for each GP value.

### Reporting summary

Further information on research design is available in the Nature Portfolio Reporting Summary linked to this article.

## Online content

Any methods, additional references, Nature Portfolio reporting summaries, source data, extended data, supplementary information, acknowledgements, peer review information; details of author contributions and competing interests; and statements of data and code availability are available at 10.1038/s41556-023-01207-8.

## Supplementary information

Reporting Summary

Supplementary Table 1 Supplementary Table 1. Yeast strains used in this study. Supplementary Table 2. Plasmids used in this study. Supplementary Table 3. GUV lipid mixes used in this study.

Supplementary Video 1 **3D ultrastructural analysis of a cell overexpressing Sct1**. Electron tomography was performed on a 250 nm resin section derived from cells overexpressing mGFP–*SCT1* from an inducible *GAL1* promoter. The sample was tilted around two axes: a-axis from −60° to +64° and b-axis from −60° to +60°, each with 1° increments. 3D animation shows a *z*-scan through a tomogram (1*z* = 1.1 nm) and a model based on the ultrastructural contours of the ER membrane network. The NE and the connected ER membranes are labelled in gold, other ER membranes in blue, PM in dark green, vacuoles in purple and LDs and vesicles in grey.

Supplementary Video 2 **3D ultrastructural analysis of a wild-type cell**. Electron tomography was performed on a 200 nm resin section derived from wild-type cells grown in SDC medium. The sample was tilted around two axes: a-axis from −60° to +60° and b-axis from −58° to +59°, each with 1° increments. 3D animation shows a *z*-scan through a tomogram (1*z* = 0.757 nm) and a model based on the ultrastructural contours of the ER membrane network. The NE and the connected ER membranes are labelled in gold, other ER membranes in blue, PM in dark green, Golgi stacks in brown, peroxisome in dark brown and LDs and vesicles in grey.

## Data Availability

The data reported in this paper are available in the main text or Supplementary Information. Source data are provided with this paper. Any additional information required to re-analyse the data reported in this paper are available from the lead contact upon request.

## Source data

Source Data Fig. 1 Statistical source data.
Source Data Fig. 1 Unprocessed immunoblots.
Source Data Fig. 2 Statistical source data.
Source Data Fig. 3 Statistical source data.
Source Data Fig. 4 Statistical source data.
Source Data Fig. 5 Statistical source data.
Source Data Fig. 6 Statistical source data.
Source Data Fig. 7 Statistical source data.
Source Data Extended Data Fig. 1 Statistical source data.
Source Data Extended Data Fig. 1 Unprocessed immunoblots.
Source Data Extended Data Fig. 2 Statistical source data.
Source Data Extended Data Fig. 2 Unprocessed immunoblots.
Source Data Extended Data Fig. 3 Statistical source data.
Source Data Extended Data Fig. 4 Statistical source data.
Source Data Extended Data Fig. 5 Statistical source data.
Source Data Extended Data Fig. 6 Statistical source data.
Source Data Extended Data Fig. 6 Unprocessed immunoblots.
