# Supplementary material for: Lipid saturation controls nuclear envelope function
Source: Nat Cell Biol. 2023 Aug 17;25(9):1290–302. doi: 10.1038/s41556-023-01207-8 (PMC10495262; doi:10.1038/s41556-023-01207-8)
Supplement: Supplementary file 1 — Reporting Summary [file 41556_2023_1207_MOESM1_ESM.pdf]

nature portfolio

Corresponding author(s): Alwin Köhler

Last updated by author(s): Jul 12, 2023

Reporting Summary

Nature Portfolio wishes to improve the reproducibility of the work that we publish. This form provides structure for consistency and transparency in reporting. For further information on Nature Portfolio policies, see our Editorial Policies and the Editorial Policy Checklist.

Statistics

For all statistical analyses, confirm that the following items are present in the figure legend, table legend, main text, or Methods section.

n/a	Confirmed	
☐	☒	The exact sample size (*n*) for each experimental group/condition, given as a discrete number and unit of measurement
☐	☒	A statement on whether measurements were taken from distinct samples or whether the same sample was measured repeatedly
☐	☒	The statistical test(s) used AND whether they are one- or two-sided<br>*Only common tests should be described solely by name; describe more complex techniques in the Methods section.*
☒	☐	A description of all covariates tested
☐	☒	A description of any assumptions or corrections, such as tests of normality and adjustment for multiple comparisons
☐	☒	A full description of the statistical parameters including central tendency (e.g. means) or other basic estimates (e.g. regression coefficient) AND variation (e.g. standard deviation) or associated estimates of uncertainty (e.g. confidence intervals)
☐	☒	For null hypothesis testing, the test statistic (e.g. *F*, *t*, *r*) with confidence intervals, effect sizes, degrees of freedom and *P* value noted<br>*Give P values as exact values whenever suitable.*
☒	☐	For Bayesian analysis, information on the choice of priors and Markov chain Monte Carlo settings
☒	☐	For hierarchical and complex designs, identification of the appropriate level for tests and full reporting of outcomes
☒	☐	Estimates of effect sizes (e.g. Cohen's *d*, Pearson's *r*), indicating how they were calculated

Our web collection on statistics for biologists contains articles on many of the points above.

Software and code

Policy information about availability of computer code

Data collection	Excluding manufacturer's software required to run the instruments described in the Methods section, no custom code was required to aquire any data in this manuscript.
Data analysis	The following software packages were used: ImageJ (version 1.4.3.67), Fiji (version 2.0.0-rc-69/1.52p), GraphPad Prism (version 9.5.1), IMOD (version 4.9.3), softWoRX (version 6.5.2), ZEISS ZEN blue (version 3.1), ZEISS ZEN Blue Desk with ZenConnect Module (version 3.3), LipotypeXplorer (version 1.4).

For manuscripts utilizing custom algorithms or software that are central to the research but not yet described in published literature, software must be made available to editors and reviewers. We strongly encourage code deposition in a community repository (e.g. GitHub). See the Nature Portfolio guidelines for submitting code & software for further information.

Data

Policy information about availability of data

All manuscripts must include a data availability statement. This statement should provide the following information, where applicable:

- Accession codes, unique identifiers, or web links for publicly available datasets
- A description of any restrictions on data availability
- For clinical datasets or third party data, please ensure that the statement adheres to our policy

The data reported in this paper are available in the main text or Supplementary Information. Source data have been provided in Source Data. Any additional information required to re-analyse the data reported in this paper are available from the lead contact upon request.

Human research participants

Policy information about studies involving human research participants and Sex and Gender in Research.

Reporting on sex and gender	Not applicable. The model is S.cerevisiae
Population characteristics	Not applicable
Recruitment	Not applicable
Ethics oversight	Not applicable

Note that full information on the approval of the study protocol must also be provided in the manuscript.

Field-specific reporting

Please select the one below that is the best fit for your research. If you are not sure, read the appropriate sections before making your selection.

☒ Life sciences ☐ Behavioural & social sciences ☐ Ecological, evolutionary & environmental sciences

For a reference copy of the document with all sections, see nature.com/documents/nr-reporting-summary-flat.pdf

Life sciences study design

All studies must disclose on these points even when the disclosure is negative.

Sample size	Reported data were generated from at least 3 biological replicates except for the Extended Figure 3q where 2 replicates were used. Sample sizes were chosen according to or exceeding standards in the field.
Data exclusions	No data were excluded except in Fig. 6a where 2 extreme outliers (out of 4477 data points) were excluded from the analysis and in Fig. 3i where outliers were identified and removed using the ROUT method (Q set to 1%) in the GraphPad Prism software.
Replication	Reported data were generated from at least 3 biological replicates except for the Extended Figure 3q where 2 replicates were used. All attempts to replicate the data were successful.
Randomization	No randomization was performed as our study was performed with liquid yeast cultures, not individual yeast cells.
Blinding	No blinding was performed as the phenotype classifications were based on rigorous quantitative criteria.

Reporting for specific materials, systems and methods

We require information from authors about some types of materials, experimental systems and methods used in many studies. Here, indicate whether each material, system or method listed is relevant to your study. If you are not sure if a list item applies to your research, read the appropriate section before selecting a response.

Materials & experimental systems

n/a	Involved in the study
☐	☒ Antibodies
☒	☐ Eukaryotic cell lines
☒	☐ Palaeontology and archaeology
☒	☐ Animals and other organisms
☒	☐ Clinical data
☒	☐ Dual use research of concern

Methods

n/a	Involved in the study
☒	☐ ChIP-seq
☒	☐ Flow cytometry
☒	☐ MRI-based neuroimaging

Antibodies

Antibodies used

Mouse monoclonal anti-mCherry (clone 1C51) (1:2000, Abcam Cat.#ab125096), Mouse monoclonal anti-GFP (clones 7.1 and 13.1) (1:2000, Roche Cat.#ab11814460001), Mouse monoclonal anti-Pgk1 (clone 22C5D8) (1:10000, Abcam Cat.#ab113687), Peroxidase AffiniPure Goat anti-Mouse IgG (polyclonal) (1:5000, Jackson ImmunoResearch Cat.#115035008), Mouse monoclonal anti-HA (clone 12CA5) (1:1000, Max Perutz Labs Monoclonal Antibody Facility).

Validation

Validation relied on positive and negative controls used in our laboratory and statements from the manufacturer's websites.

Mouse monoclonal anti-mCherry
Suitable for: WB, ICC/IF, IHC-P
Species reactivity: Species independent

Mouse monoclonal anti-GFP
Suitable for: WB, Immunoprecipitation, Immunostaining
Species reactivity: Species independent

Mouse monoclonal anti-Pgk1
Suitable for: WB
Species reactivity: Saccharomyces cerevisiae

Peroxidase AffiniPure Goat anti-Mouse IgG
Suitable for: WB, ELISA, immunohisto/cytochemistry
Species reactivity: Species independent

Mouse monoclonal anti-HA
Suitable for: WB
Species reactivity: Species independent
