# Supplementary figures and images for: Lipid saturation controls nuclear envelope function
Source: Nat Cell Biol. 2023 Aug 17;25(9):1290–302. doi: 10.1038/s41556-023-01207-8 (PMC10495262; doi:10.1038/s41556-023-01207-8)

Fig. 1d

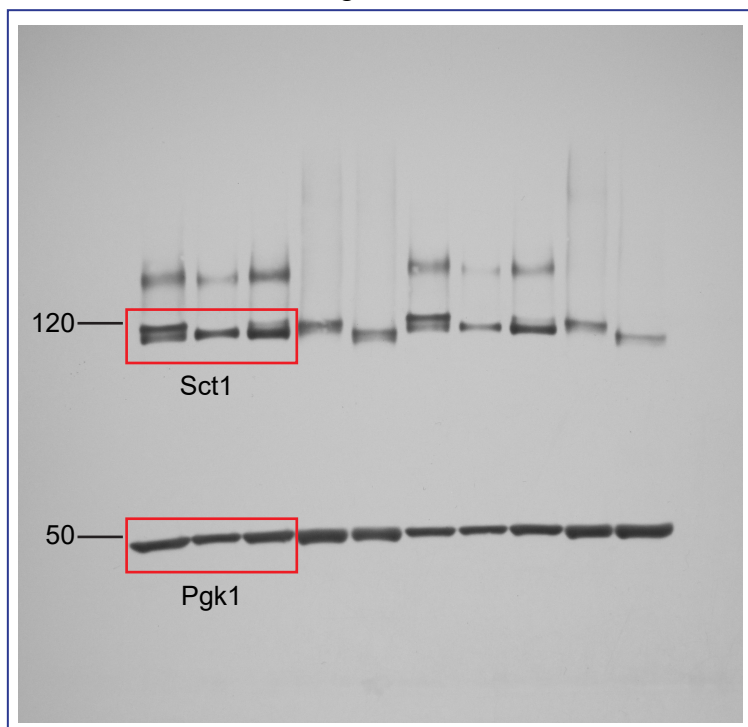

Supplement: Source Data Fig. 1 — Unprocessed immunoblots. [file 41556_2023_1207_MOESM6_ESM.pdf]

Extended Data Fig. 1f

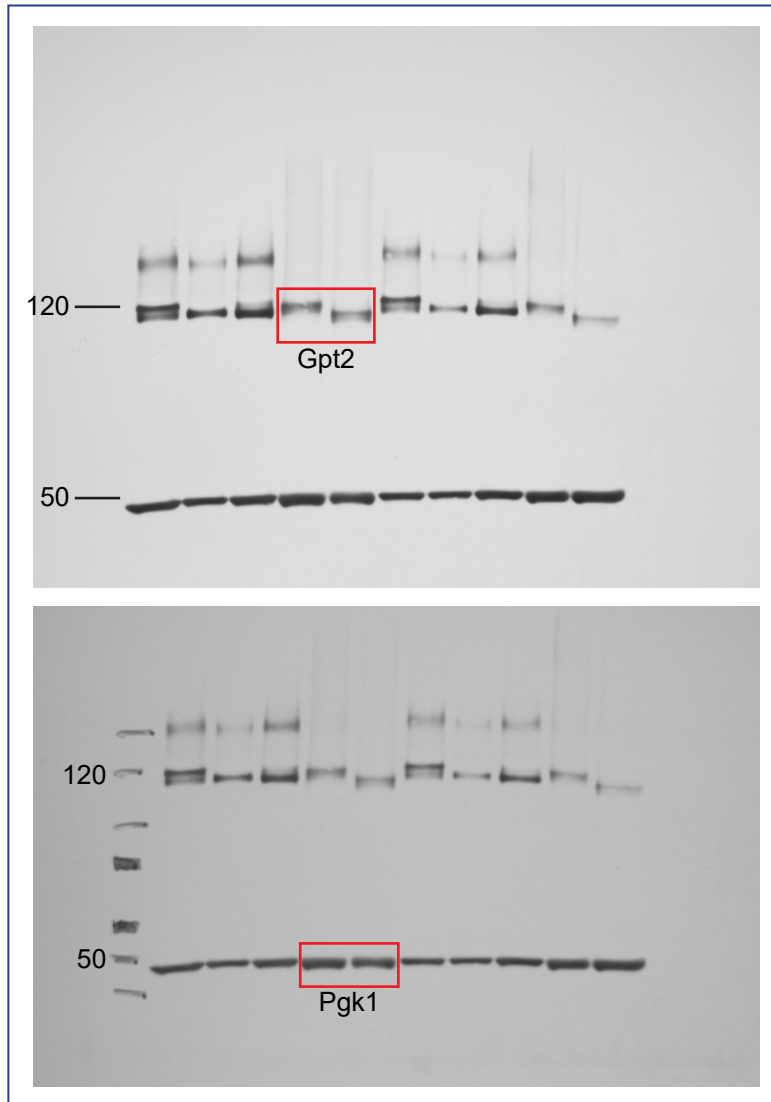

Supplement: Source Data Extended Data Fig. 1 — Unprocessed immunoblots. [file 41556_2023_1207_MOESM14_ESM.pdf]

Extended Data Fig. 2a

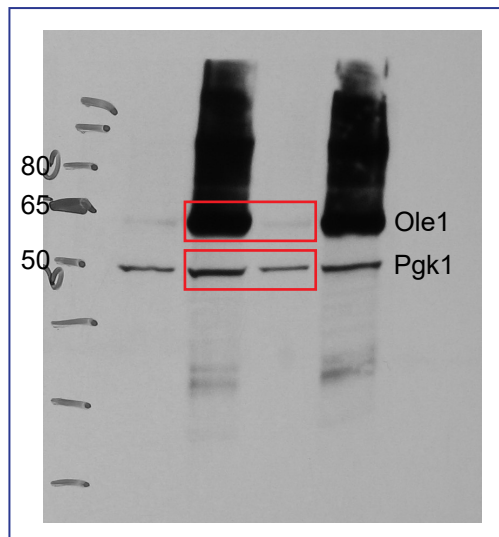

Extended Data Fig. 2b

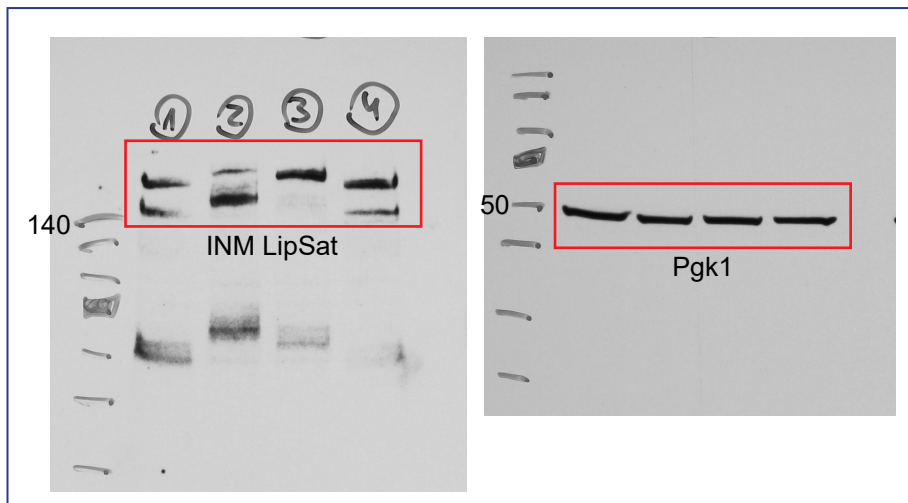

Extended Data Fig. 2e

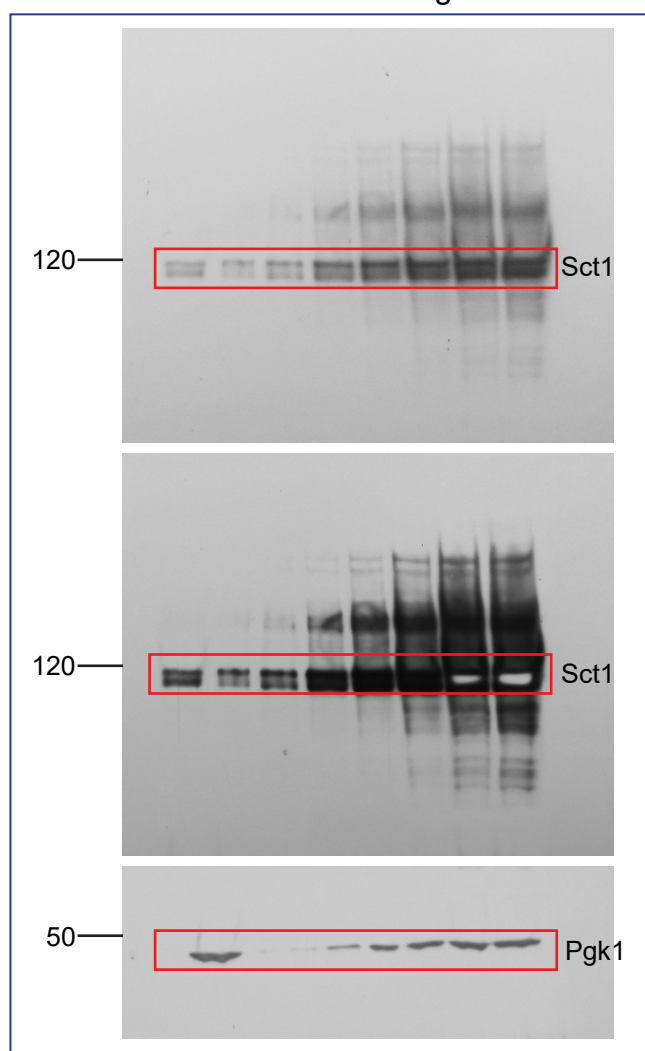

Extended Data Fig. 2f

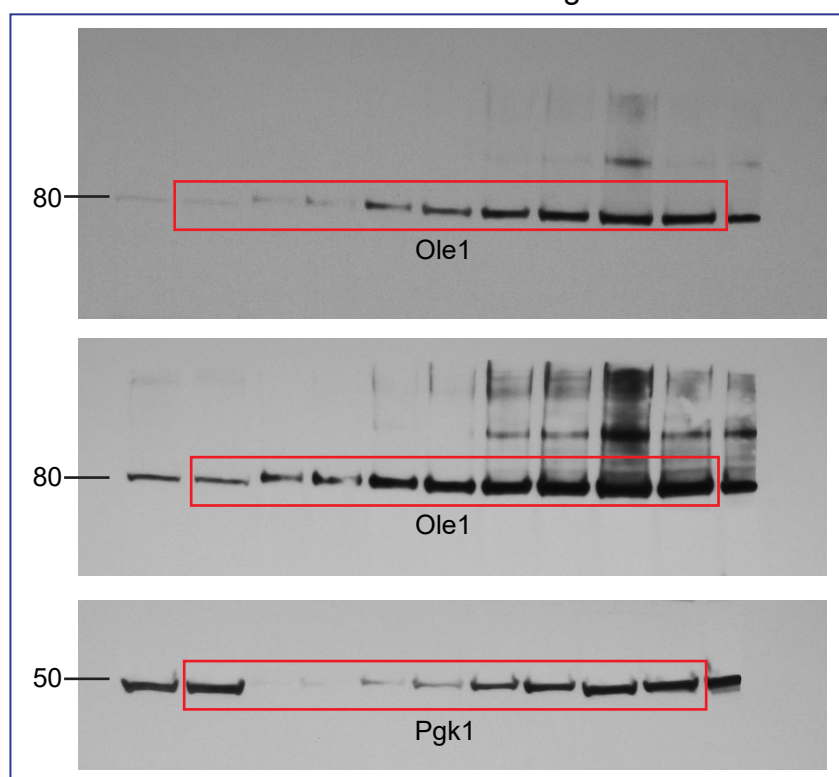

Supplement: Source Data Extended Data Fig. 2 — Unprocessed immunoblots. [file 41556_2023_1207_MOESM16_ESM.pdf]

Extended Data Fig. 6e

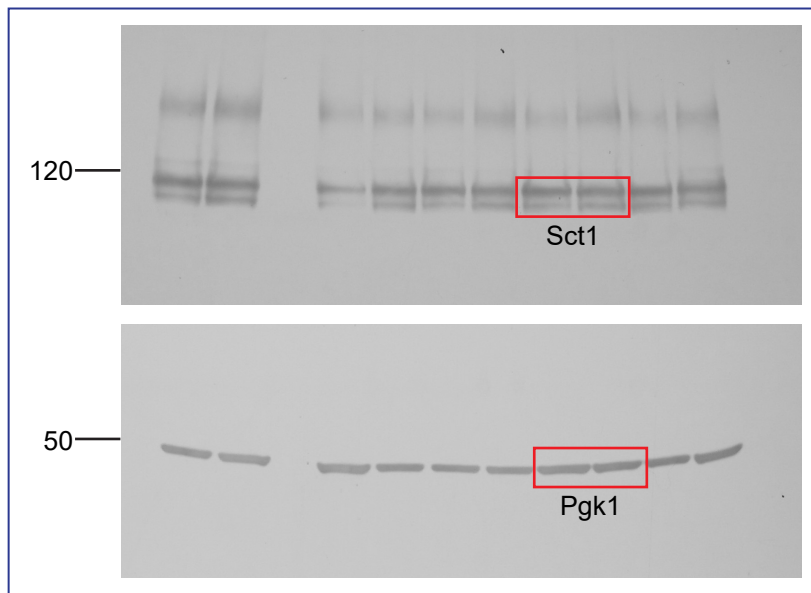

Supplement: Source Data Extended Data Fig. 6 — Unprocessed immunoblots. [file 41556_2023_1207_MOESM21_ESM.pdf]
